# CsrA Enhances Cyclic-di-GMP Biosynthesis and Yersinia pestis Biofilm Blockage of the Flea Foregut by Alleviating Hfq-Dependent Repression of the *hmsT* mRNA

**DOI:** 10.1128/mBio.01358-21

**Published:** 2021-08-03

**Authors:** Amelia R. Silva-Rohwer, Kiara Held, Janelle Sagawa, Nicolas L. Fernandez, Christopher M. Waters, Viveka Vadyvaloo

**Affiliations:** a Paul G. Allen School for Global Health, Washington State University, Pullman, Washington, USA; b Department of Microbiology and Molecular Genetics, Michigan State University, East Lansing, Michigan, USA; University of Michigan-Ann Arbor

**Keywords:** *Yersinia pestis*, *Xenopsylla cheopis* fleas, carbon storage regulator, c-di-GMP

## Abstract

Plague-causing Yersinia pestis is transmitted through regurgitation when it forms a biofilm-mediated blockage in the foregut of its flea vector. This biofilm is composed of an extracellular polysaccharide substance (EPS) produced when cyclic-di-GMP (c-di-GMP) levels are elevated. The Y. pestis diguanylate cyclase enzymes HmsD and HmsT synthesize c-di-GMP. HmsD is required for biofilm blockage formation but contributes minimally to *in vitro* biofilms. HmsT, however, is necessary for *in vitro* biofilms and contributes to intermediate rates of biofilm blockage. C-di-GMP synthesis is regulated at the transcriptional and posttranscriptional levels. In this, the global RNA chaperone, Hfq, posttranscriptionally represses *hmsT* mRNA translation. How c-di-GMP levels and biofilm blockage formation is modulated by nutritional stimuli encountered in the flea gut is unknown. Here, the RNA-binding regulator protein CsrA, which controls c-di-GMP-mediated biofilm formation and central carbon metabolism responses in many Gammaproteobacteria, was assessed for its role in Y. pestis biofilm formation. We determined that CsrA was required for markedly greater c-di-GMP and EPS levels when Y. pestis was cultivated on alternative sugars implicated in flea biofilm blockage metabolism. Our assays, composed of mobility shifts, quantification of mRNA translation, stability, and abundance, and epistasis analyses of a *csrA hfq* double mutant strain substantiated that CsrA represses *hfq* mRNA translation, thereby alleviating Hfq-dependent repression of *hmsT* mRNA translation. Additionally, a *csrA* mutant exhibited intermediately reduced biofilm blockage rates, resembling an *hmsT* mutant. Hence, we reveal CsrA-mediated control of c-di-GMP synthesis in Y. pestis as a tiered, posttranscriptional regulatory process that enhances biofilm blockage-mediated transmission from fleas.

## INTRODUCTION

Yersinia pestis evolved clonally from the gastrointestinal pathogen Yersinia pseudotuberculosis to be transmitted via flea bite (1–3). Within the flea gut, Y. pestis derives its nutrition from the bloodmeal and by-products of blood digestion to multiply and form a cohesive biofilm (4, 5). This enables development of a biofilm-mediated blockage of the flea foregut. Blockage facilitates regurgitation of bacteria back into the flea bite site of the mammalian host to cause plague (3, 6).

Biofilms are multicellular bacterial communities encased in self-produced extracellular polymeric substances (EPS). Poly-β-1,6-*N-acetyl*-d-glucosamine exopolysaccharides (PNAG) comprise the Y. pestis EPS and are synthesized and exported by the gene products of the *hmsHFRS* operon (7, 8). The *hmsHFRS* operon is highly transcribed at flea optimal temperatures of ≤26°C, and the gene products are produced at elevated c-di-GMP levels (9, 10). In many bacteria, the planktonic/sessile and biofilm-producing states are directed by low or high levels of c-di-GMP, respectively (11–13). In the case of Y. pestis, three of the four discrete genetic changes that confer the trait of biofilm-mediated blockage transmissibility to this pathogen occur in loci involved in c-di-GMP metabolism (14, 15).

C-di-GMP is synthesized by diguanylate cyclases (DGCs) and degraded by phosphodiesterase (PDE) enzymes. Two DGCs, encoded by *hmsT* and *hmsD*, and one PDE encoded by *hmsP*, modulate c-di-GMP synthesis and hydrolysis, respectively, and are involved in biofilm production in Y. pestis (16–20). Although *hmsT* and *hmsD* have comparable transcript levels *in vitro* and in fleas (20), HmsD is predominantly involved in c-di-GMP synthesis in the flea. An *hmsD* mutant is severely impaired in biofilm-mediated flea blockage but exhibits only small reductions in *in vitro* biofilm formation (19, 20). Conversely, HmsT is the predominant DGC for *in vitro* biofilms. An *hmsT* mutant produces little to no biofilm *in vitro* and exhibits intermediate flea blockage rates (17, 20, 21). HmsT protein abundance is regulated at the transcriptional and posttranscriptional levels, while HmsD protein abundance is regulated posttranslationally. Hfq, the global RNA binding protein, posttranscriptionally represses *hmsT* mRNA (22, 23), while the Rcs phosphorelay system response regulator protein, RcsB, inhibits *hmsT* gene expression (24). HmsD is part of the HmsCDE locus encoding a tripartite signaling system, wherein HmsD is inversely modulated by HmsC and HmsE proteins in response to specific environmental stimuli (16, 19).

How Y. pestis integrates nutritional stimuli encountered in the flea gut to modulate c-di-GMP synthesis is unknown. The carbon storage regulator protein, CsrA, therefore was of interest because it posttranscriptionally coordinates physiological adaptations to changing nutritional environments in many bacteria. Additionally, the *csrA* gene is highly transcribed in Y. pestis blocked fleas (4). CsrA is a widely conserved global RNA binding protein in Gammaproteobacteria species, where it functions to modulate central carbon metabolism, cellular development, and pathogenesis and exhibits well-defined involvement in regulating biofilm formation (25–36). CsrA binds to the GGA motifs within 5′ untranslated regions (5′UTR) of target mRNAs to alter their translation (37–41). Two noncoding RNAs (ncRNAs), CsrB and CsrC, containing numerous CsrA binding motifs, sequester and antagonize CsrA activity by competing for binding with target mRNAs (30).

CsrA primarily posttranscriptionally represses mRNA targets that activate biofilm formation in bacteria (25, 31, 36, 42, 43). However, CsrA positively regulates *in vitro* biofilm production in Y. pestis by an undefined mechanism (44). Here, we sought to determine if CsrA has a role in the physiologically relevant context of *in vivo* biofilm-mediated flea blockage. We determined that CsrA promoted *in vitro* biofilm production more stringently when alternative sugars implicated in flea biofilm formation were supplemented in the culture medium. Additionally, we identified that the mechanism by which CsrA positively regulated biofilm production was through translational inhibition of the *hfq* mRNA, which posttranscriptionally represses the *hmsT* mRNA required for c-di-GMP biosynthesis. Lastly, we determined that Y. pestis CsrA is needed for robust biofilm-mediated blockage of the transmission-proficient rat flea, Xenopsylla cheopis.

## RESULTS

### CsrA positively regulates *in vitro* EPS and intracellular c-di-GMP levels.

Biofilm formation of a Y. pestis *csrA* mutant is impaired during growth on alternative carbon sources (e.g., K-gluconate) versus the primary carbon source glucose (44). The alternative sugars ribose and galactose appear to be primarily catabolized by Y. pestis during flea blockage (4, 5). Hence, we tested if biofilm EPS formation in a *csrA* mutant is more drastically impaired in these biologically relevant sugars versus glucose. An assay based on the specificity of Congo red (CR) dye to polysaccharides (17, 45) was used to allow direct comparison of EPS production from different carbon sources through normalization by bacterial biomass. The assay media were HIB, a rich routine culture medium, and the chemically defined medium TMH (46), supplemented with either glucose (TMH-glu), ribose (TMH-rib), or galactose (TMH-gal).

**FIG 1:**
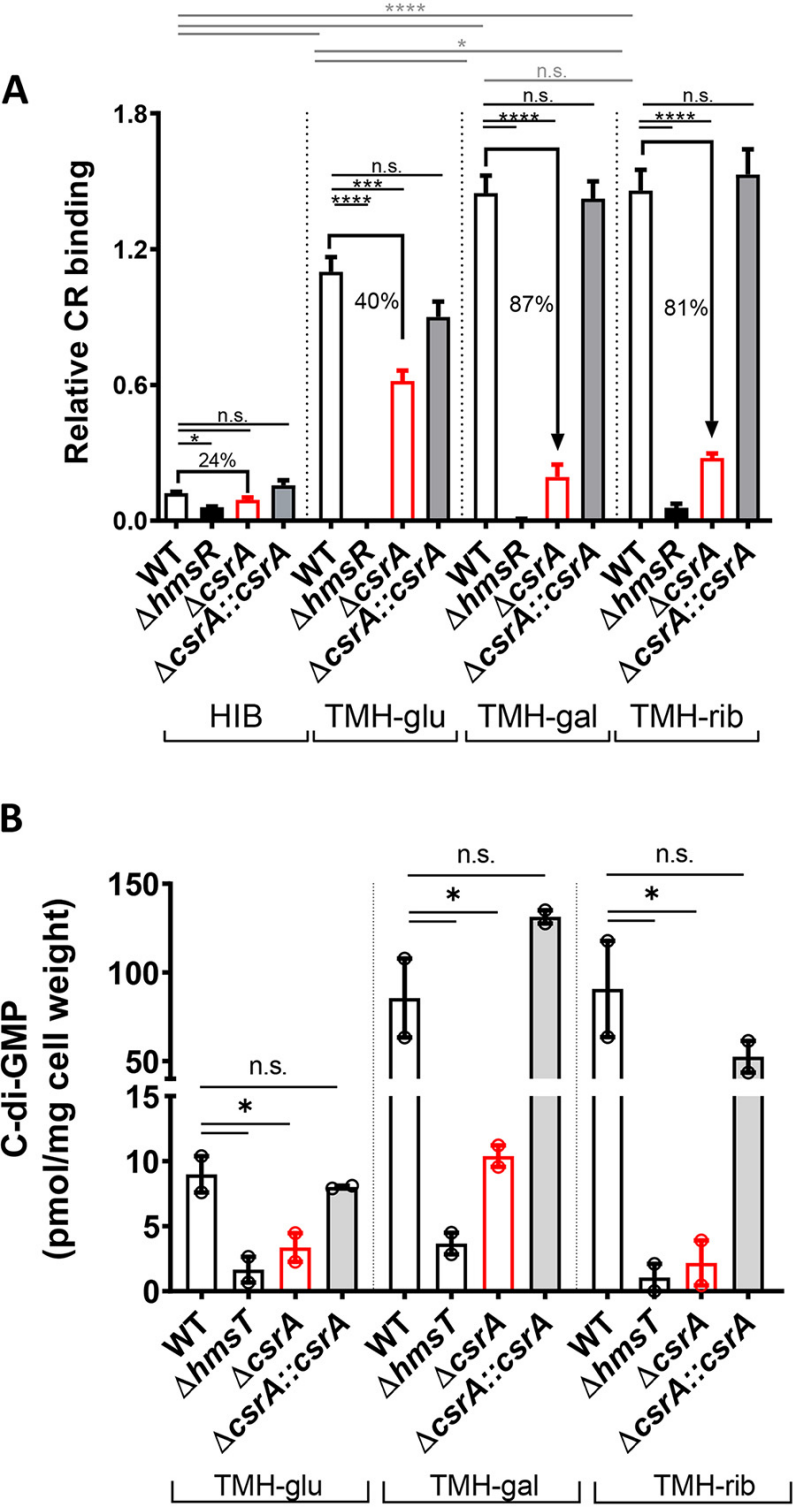
CsrA is required for EPS production and c-di-GMP synthesis. (A) Congo red (CR) binding assays were used to quantify EPS production of strains cultured in HIB or TMH supplemented with 0.2% of glucose (TMH-glu), galactose (TMH-gal), or ribose (TMH-rib). Error bars represent the means ± standard errors of the means (SEM) of bound CR samples from 3 to 6 independent experiments. (B) C-di-GMP was extracted from strains grown in TMH-glu, TMH-gal, or TMH-rib. Means of two independent experiments are shown. Statistical significance was determined using one-way analysis of variance (ANOVA) with Dunnett’s multiple comparisons posttest for each medium type (black lines) or to compare the WT strain across media (gray lines) (*, *P* < 0.05; ***, *P*,  0.0005; ****, *P* < 0.0001; n.s., not significant).

A *csrA* mutant (Δ*csrA*) generated previously (44) in the avirulent epidemic KIM6+ strain background, with an isogenic wild-type (WT) parent strain and a *cis*-complemented *csrA* mutant strain (Δ*csrA*::*csrA*), were tested for EPS production. EPS production by the WT strain in HIB was >10-fold less than that in TMH medium regardless of the carbon source (Fig. 1A). EPS production in the WT strain was significantly lower in TMH-glu versus TMH-gal and TMH-rib. Under all conditions, a Δ*hmsR* strain, used as a negative control as it is unable to produce EPS, displayed little to no CR binding. Compared to the WT strain, the Δ*csrA* strain had significantly reduced EPS levels under all conditions, a phenotype that was restored in the Δ*csrA*::*csrA* strain. Mean reduction in EPS levels for the Δ*csrA* strain relative to the WT strain were 24% and 40% in HIB and TMH-glu and 87% and 81% in TMH-rib and TMH-gal, respectively.

To assess if EPS levels in the Δ*csrA* strain correlated with intracellular c-di-GMP pools, we quantified c-di-GMP in strains grown in TMH-glu, TMH-gal, and TMH-rib. The c-di-GMP levels of the WT strain ranged between 7.6 and 10.4, 63.4 to 107.8, and 63.6 to 117.8 pmol/mg cell weight in TMH-glu, TMH-gal, and TMH-rib, respectively (Fig. 1B). Under all conditions, a Δ*hmsT* strain, dysfunctional in c-di-GMP synthesis (19), produced little to no c-di-GMP, as expected (18, 19, 22). Compared to the WT strain, the Δ*csrA* strain had 2.7-, 8.2-, and 42-fold mean reduction in c-di-GMP levels in TMH-glu, TMH-gal, and TMH-rib, respectively. The Δ*csrA*::*csrA* strain exhibited c-di-GMP levels within ranges displayed by the WT strain. Therefore, biofilm and c-di-GMP production in TMH-gal and TMH-rib was highly dependent on functional CsrA. However, TMH-gal was selected for the next experiments to allow for comparison with published studies (47).

### CsrA promotes translation of the *hmsT* mRNA.

CsrA alters translation rates of mRNAs encoding enzymes for c-di-GMP synthesis or degradation, thereby altering c-di-GMP and EPS production in other bacteria (25, 35, 42, 43). Willias et al. (44) proposed that CsrA targets the *hmsP* and/or *hmsT* mRNAs to reduce c-di-GMP levels in the Δ*csrA* strain and identified putative CsrA binding sites in the 5′ UTRs of these transcripts. Our experiments described above support this idea, since we showed reduced c-di-GMP levels in the Δ*csrA* strain.

**FIG 2:**
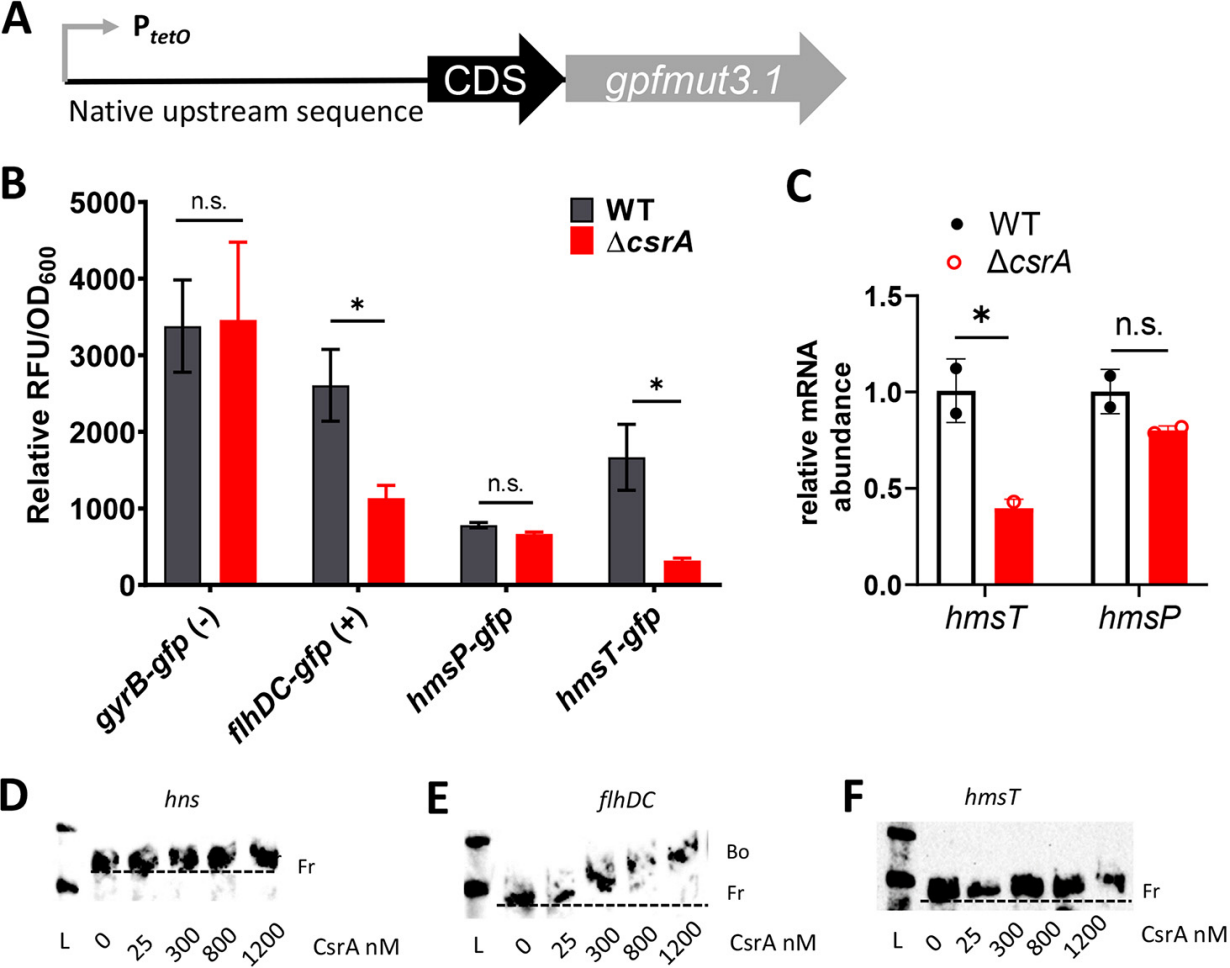
CsrA does not directly regulate *hmsT* mRNA translation. (A) Schematic of the inducible GFP fusion reporters in which the native upstream untranslated sequences and the first 9 or 10 codons of *gyrB* (negative control), *flhDC* (positive control), *hmsP*, and *hmsT* were fused in frame to *gfpmut3.1* (*gfp*) and the anhydrotetracyline (ATc)-inducible promoter P_tetO_ in the WT and Δ*csrA* strains. (B) Posttranscriptional fusion reporter strains grown in TMH-gal to log phase were induced with ATc. At 3 h postinduction, the relative fluorescent units (RFU) and OD_600_ were measured. Uninduced RFU/OD_600_ values were subtracted from induced RFU/OD_600_ values to compare between strains. Error bars represent means ± SEM from three independent experiments. Statistical significance was determined by an unpaired *t* test (*, *P* < 0.05; n.s., not significant). (C) Steady-state transcript levels of *hmsP* and *hmsT* were compared between the WT and Δ*csrA* strains. Means ± SD from two independent experiments are shown. Statistical significance was determined with a Student's *t* test (*, *P* < 0.05; n.s., not significant). For gel mobility shift assays, 0.8 nM 3′biotin end-labeled *hns* (negative control) (D), *flhDC* (positive control) (E), or *hmsT* (F) probes were incubated with increasing concentrations of purified CsrA-His_6_. Kerafast biotinylated sRNA ladder (L), free (Fr), and bound (Bo) species are indicated. A broken line indicates migration of Fr labeled probe. One representative of two independent experiments is shown.

To determine if translation of the *hmsP* and *hmsT* mRNAs are CsrA dependent, we constructed posttranscriptional green fluorescent protein (GFP) fusion reporters. The 5′ UTR plus predicted CsrA binding motifs (44) were engineered in-frame to the coding sequence (CDS) of *gfpmut3.1* (Fig. 2A) and an inducible promoter, P_tetO_. The use of the P_tetO_ promoter was intended to uncouple transcription from control by growth phases, environmental signals, or transcription factors. The 5′ UTR of *flhDC* was used as a positive control because it is a validated target of CsrA in Y. pseudotuberculosis (48) and shares 100% nucleotide identity with the 5′ UTR of Y. pestis *flhDC* mRNA. CsrA is identical between Y. pestis and Y. pseudotuberculosis; thus, Y. pestis CsrA was expected to bind the *flhDC* transcript. The 5′ UTR of housekeeping gene *gyrB* that lacks CsrA binding motifs was used as a negative control.

GFP reporter fusion constructs were transformed into the WT and Δ*csrA* strains. Strains were grown in TMH-gal and fluorescence recorded at 3 h postinduction, when the greatest GFP induction was achieved for each construct (data not shown). As expected, GFP expression between the WT and Δ*csrA* strains from the *gyrB-gfp* reporter was comparable but was significantly reduced in the Δ*csrA* strain with the *flhDC-gfp* reporter (Fig. 2B). No significant difference in GFP expression between the WT and Δ*csrA* strains was noted for the *hmsP-gfp* reporter. However, significant reduction in GFP expression occurred in the *hmsT-gfp* reporter in the Δ*csrA* strain compared to the WT strain, suggesting that the *hmsT* mRNA translation was CsrA dependent.

Next, to determine if decreased *hmsT* mRNA translational levels in the Δ*csrA* strain are a result of decreased *hmsT* mRNA levels, we evaluated steady-state levels of the *hmsT* mRNA in the WT and Δ*csrA* strains using reverse transcription-quantitative PCR (RT-qPCR). We included an evaluation of the *hmsP* mRNA as a negative control, as *hmsP* mRNA translational levels were unaffected by CsrA. The relative steady-state mRNA levels of the *hmsT* mRNA were significantly lower in the Δ*csrA* strain than the WT strain, while the *hmsP* mRNA levels were similar between these strains (Fig. 2C). These data suggested that *hmsT* mRNA abundance was CsrA dependent.

### CsrA does not bind directly to the *hmsT* mRNA.

To determine if CsrA regulation of the *hmsT* mRNA results from direct binding of CsrA, RNA electrophoretic mobility shift assays (REMSAs) were conducted. A transcript of the *hmsT* mRNA containing the same region as that in the *hmsT-gfp* reporter fusion was used to examine the interaction of the *hmsT* mRNA 5′ UTR and CsrA. The *hns* mRNA that does not bind to CsrA was used as a negative control, and the 5′ UTR of *flhDC* served as a positive control (48). As previously reported (48), the labeled *hns* probe did not shift with increasing concentrations of CsrA-His_6_ (Fig. 2D). As expected, a shift occurred for the labeled *flhDC* probe at increasing concentrations of CsrA-His_6_ (Fig. 2E). No shift was seen for the labeled *hmsT* probe with increasing concentrations of CsrA-His_6_ (Fig. 2F), indicating that CsrA was not able to bind directly to the *hmsT* mRNA. These results strongly suggested that CsrA indirectly regulated *hmsT* mRNA translation.

### CsrA binds specifically to the *hfq* mRNA.

**FIG 3:**
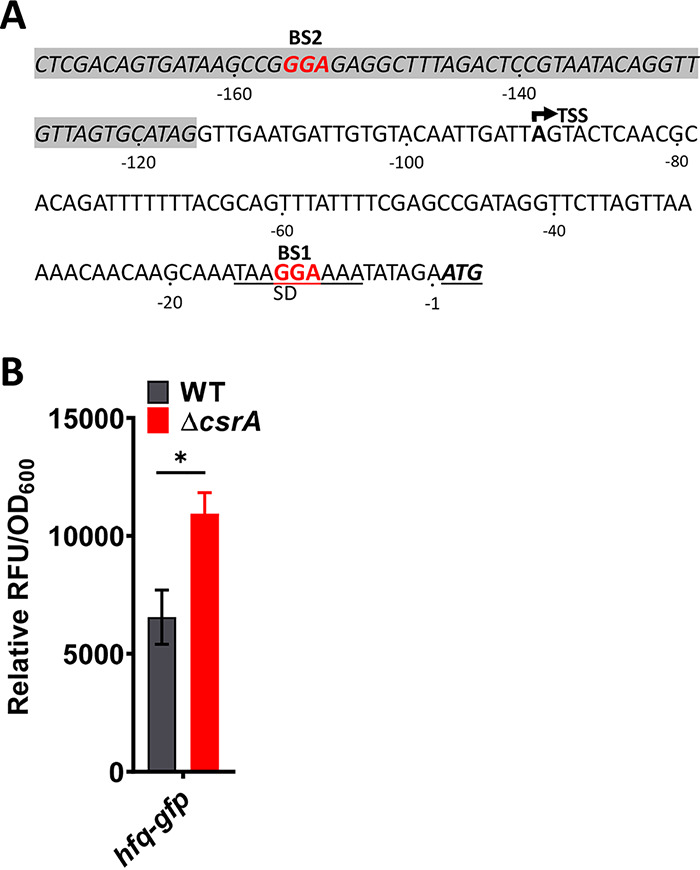
CsrA negatively regulates *hfq* mRNA translation. (A) Nucleotide sequence of the 3′ end of the *miaA* gene (shaded gray) and the intercistronic region *miaA*-*hfq* genes plus ATG start of the *hfq* mRNA. The Shine-Dalgarno (SD) sequence and the ATG start codon for the *hfq* mRNA coding region are boldfaced and underlined. GGA motifs of the two putative CsrA binding sites are in red. The transcriptional start site (TSS) from the immediate upstream promoter is depicted by an arrow. (B) Posttranscriptional fusion reporter plasmids composing the upstream sequence and the first 9 codons of *hfq* fused to *gfpmut3.1* (*gfp*), and the P_tetO_ promoter was used in the WT and Δ*csrA* strains. Error bars represent mean ± SEM relative RFU/OD_600_ from four independent experiments.

The mRNAs of known negative regulators of *hmsT*, RcsB or Hfq, may instead be targeted by CsrA. Indeed, CsrA orthologs of the plant pathogen Erwinia amylovora and E. coli repress mRNAs of Y. pestis orthologs of *rcsB* (33) and *hfq* (49), respectively. Both *rcsB* and *hfq* genes are part of polycistronic operons (33, 49–51). CsrA can target downstream genes within polycistronic operons by binding to CsrA binding motifs located in the 3′ end of the upstream gene (52). Therefore, to determine if Y. pestis *rcsB* and *hfq* mRNAs are candidate mRNA targets of CsrA, a position matrix scan (44) was applied to the 5′ UTR of *rcsB* and *hfq* mRNAs to identify putative CsrA binding sites. Up to −250 bases upstream from the translational initiation codon were queried, in keeping with ranges for to-date validated CsrA targeted 5′ UTRs (36, 53, 54). Two potential binding sites were found in the *hfq* transcript (Fig. 3A; see also Fig. S1 in the supplemental material); one overlapped the Shine-Dalgarno (SD) sequence of the *hfq* mRNA (BS1) and the other occurred in a stem-loop, spanning the −151 to −159 nucleotide sequence located at the 3′ end of the upstream gene, *miaA* (BS2). The *rcsB* leader region contained no potential CsrA binding sites (data not shown). To experimentally test if *hfq* mRNA translation was CsrA dependent, we first generated an *hfq-gfp* posttranscriptional fusion reporter containing both putative CsrA binding sites. The GFP reporter expression from the *hfq*-*gfp* reporter in TMH-gal medium was compared between the WT and Δ*csrA* strains (Fig. 3B). Expression was significantly higher in the Δ*csrA* strain, suggesting that CsrA repressed *hfq* mRNA translation.

10.1128/mBio.01358-21.1 FIG S1 Secondary structure of the *hfq* 5′ untranslated region as predicted by the Mfold algorithm (Markham and Zuker, Nucleic Acids Res **33**:577–581, 2005). The positions of the CsrA binding GGA sites are in green. The ATG start of the *hfq* gene is highlighted by a light blue box. BS, binding site. Download FIG S1, TIF file, 2.1 MB. Copyright © 2021 Silva-Rohwer et al. 2021 Silva-Rohwer et al. https://creativecommons.org/licenses/by/4.0/ This content is distributed under the terms of the Creative Commons Attribution 4.0 International license.

**FIG 4:**
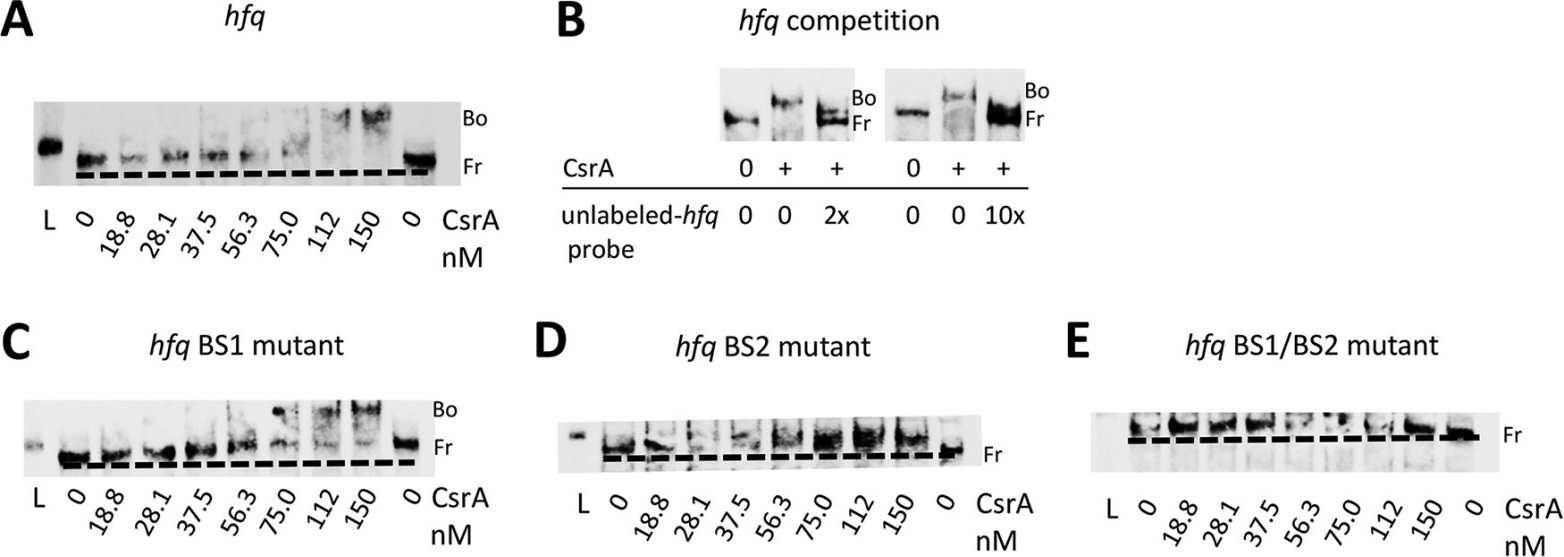
CsrA binds to two GGA binding sites in the 5′ UTR of *hfq* mRNA. (A) 3′Biotin end-labeled *hfq* probe (0.8 nM) and increasing concentrations of purified CsrA-His_6_ were coincubated. Kerafast biotinylated sRNA ladder (L), free (Fr), and bound (Bo) species are indicated. A broken line indicates migration of Fr labeled probe. (B) For mobility shift competition assays, labeled *hfq* probe was coincubated with unlabeled *hfq* probe at 2 (1.6 nM) or 10 (8 nM) times more than the labeled *hfq* probe and 112.5 nM CsrA-His_6_. (C, D, and E) Binding site mutant mobility shift assays were performed as described for panel A, except the *hfq*-labeled probes contained a mutation at BS1 (C), BS2 (D), or BS1/BS2 (E). One representative of two independent experiments is shown.

To determine if CsrA binds directly to the *hfq* mRNA, REMSAs were conducted. A minor shift in the labeled *hfq* probe was noted at 75 nM CsrA-His_6_, but at 150 nM CsrA-His_6_ the entire complex had shifted (Fig. 4A). Competitive binding assays verified that CsrA binding to the *hfq* mRNA was specific because, when competed with 2- and 10-fold excess unlabeled *hfq* probe, the labeled *hfq* probe shifted only partially or not at all, respectively (Fig. 4B).

To determine if CsrA binds to the identified GGA sites, we generated labeled probes with CC substitutions for the GG nucleotides in the GGA motif for BS1 (*hfq* BS1 mutant), CCC substitutions for the GGA motif at BS2 (*hfq* BS2 mutant) alone, and the respective aforementioned substitutions at both sites (*hfq* BS1/BS2 mutant). All mutant probes showed reduced binding to CsrA-His_6_ but at varied levels (Fig. 4C to E). For the *hfq* BS1 mutant probe, a small effect on binding to CsrA-His_6_ was noted by the presence of two complexes, one that had not shifted and the other that had shifted at ≥112 nM CsrA-His_6_ (Fig. 4C). The *hfq* BS2 mutant probe (Fig. 4D) and *hfq* BS1/BS2 mutant probe (Fig. 4E) both exhibited a complete loss of binding to CsrA-His_6_, because no shift was noted. Y. pestis *hfq* mRNA can also be transcribed from a promoter immediately upstream of its translational start codon. In this case, the *hfq* transcript composes a 90-nucleotide 5′ UTR sequence with only BS1 present (50). Additionally, a shorter GFP reporter fusion construct representative of this shorter 5′ UTR (−115 through +27 nucleotides in relation to the ATG start) had increased GFP expression in the Δ*csrA* strain compared to that of the WT strain (Fig. S2). Thus, BS1 and BS2 are authentic CsrA binding sites.

10.1128/mBio.01358-21.2 FIG S2 CsrA negatively regulates translation of the *hfq* mRNA originating from its immediate upstream promoter. The upstream sequence and partial coding sequence of *hfq* (−116 through +27 bp relative to the start codon) was fused in frame to *gfpmut3.1* (*gfp*), and an ATc-inducible promoter was used to drive expression in WT and Δ*csrA* strains. Error bars represent mean ± SEM relative RFU/OD_600_ from four independent experiments. Download FIG S2, TIF file, 0.3 MB. Copyright © 2021 Silva-Rohwer et al. 2021 Silva-Rohwer et al. https://creativecommons.org/licenses/by/4.0/ This content is distributed under the terms of the Creative Commons Attribution 4.0 International license.

### CsrA blocks translation but does not accelerate the decay rate of the *hfq* mRNA.

**FIG 5:**
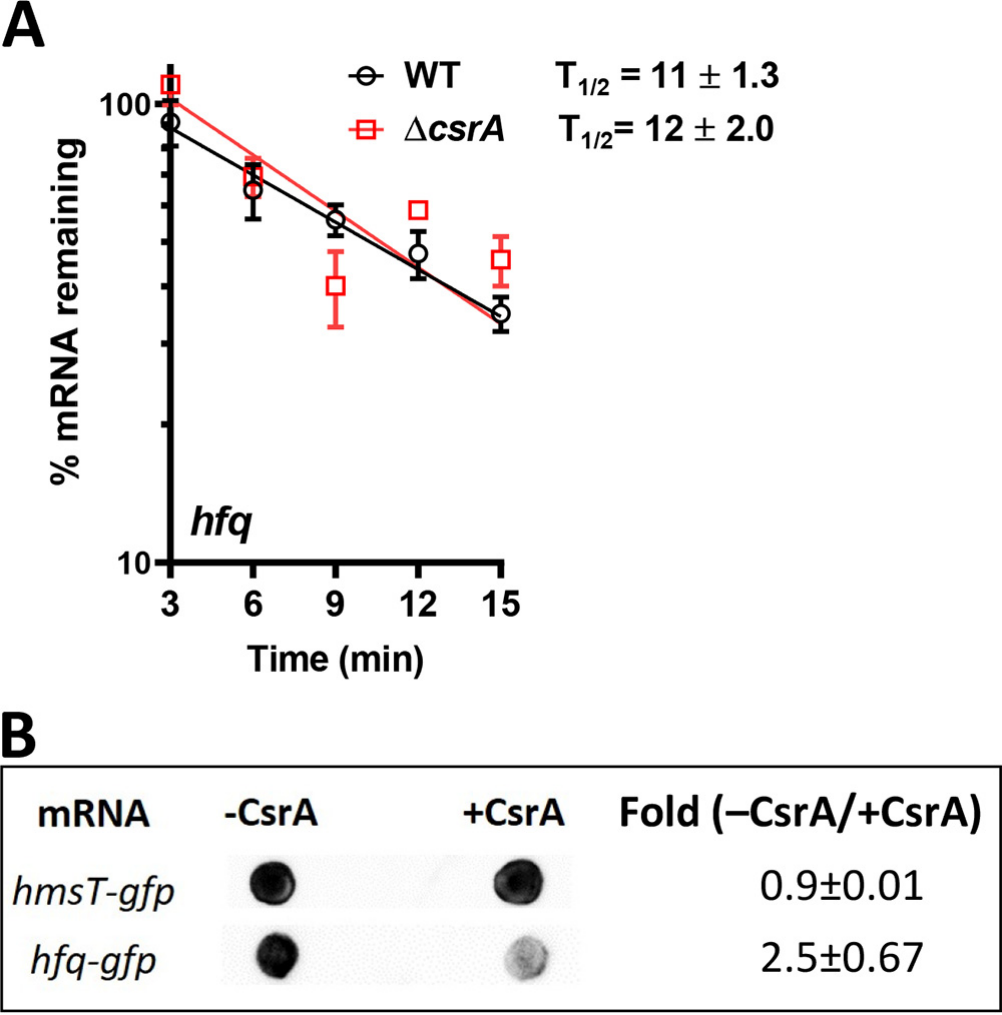
mRNA stability and translation of the *hfq* mRNA. (A) The WT and Δ*csrA* strains were grown to log phase in TMH-gal, and RNA was isolated from samples taken at 0 min (prerifampin) and after the addition of 400 μg/ml rifampin. Relative *hfq* mRNA levels remaining at the indicated time points were quantified using RT-qPCR. The amount of *hfq* mRNA in each strain at 0 min relative to rifampin addition was set to 100%. The percent mRNA remaining thereafter was plotted versus time as semilogarithmic graphs. The mean ± SEM percent mRNA from four independent experiments is shown. (B) *In vitro* translational assays were performed with the PURExpress kit using translational fusions transcripts of *hfq-gfp* and *hmsT-gfp* (negative control) expressed from a T7 promoter. The mean ± SD fold change in HmsT-GFP and Hfq-GFP signal between samples in the presence or absence of CsrA was derived from three technical replicates of the immunodot blot. One representative dot blot is shown.

CsrA may decrease *hfq* mRNA translation by accelerating its decay rate, blocking its translation by preventing ribosome binding, or by facilitating premature transcription termination (55). To test if CsrA alters stability of the *hfq* mRNA, we determined the half-life of this transcript in the WT and Δ*csrA* strains after addition of rifampin to prevent transcription initiation. The mean (± standard deviation [SD]) half-life of the *hfq* mRNA (Fig. 5A) was similar between the WT (12 ± 2.0 min) and Δ*csrA* (11.3 ± 1.3 min) strains. Therefore, CsrA binding does not destabilize the *hfq* mRNA.

To determine if *hfq* mRNA translational repression results from CsrA binding and preventing translation, we performed *in vitro* cell-free translational assays. We initially utilized an mRNA template containing the full-length sequence of the *hfq* gene engineered with a Flag tag preceding the stop codon. However, similar to a previous report using a full-length E. coli *hfq* gene template in cell-free translational assays (49), multiple bands were observed on immunoblots due to incomplete denaturation of the hexameric Hfq protein. Hence, mRNA templates derived from the *hfq-gfp* and *hmsT*-*gfp* (negative control) reporters described above were used. Similar amounts of the HmsT-GFP protein were synthesized in the presence and absence of CsrA (Fig. 5B), as expected if CsrA did not directly bind and alter translational levels of the HmsT mRNA. However, translation of the *hfq*-*gfp* transcript was inhibited in the presence of CsrA (Fig. 5B), as reflected by the 2.5 (±SD, 0.67) greater levels of Hfq-GFP protein in the absence of CsrA relative to its presence. This outcome supported that CsrA binds directly to and prevents translation of the *hfq* mRNA.

### CsrA represses *hfq* mRNA translation, facilitating derepression of *hmsT* mRNA translation.

**FIG 6:**
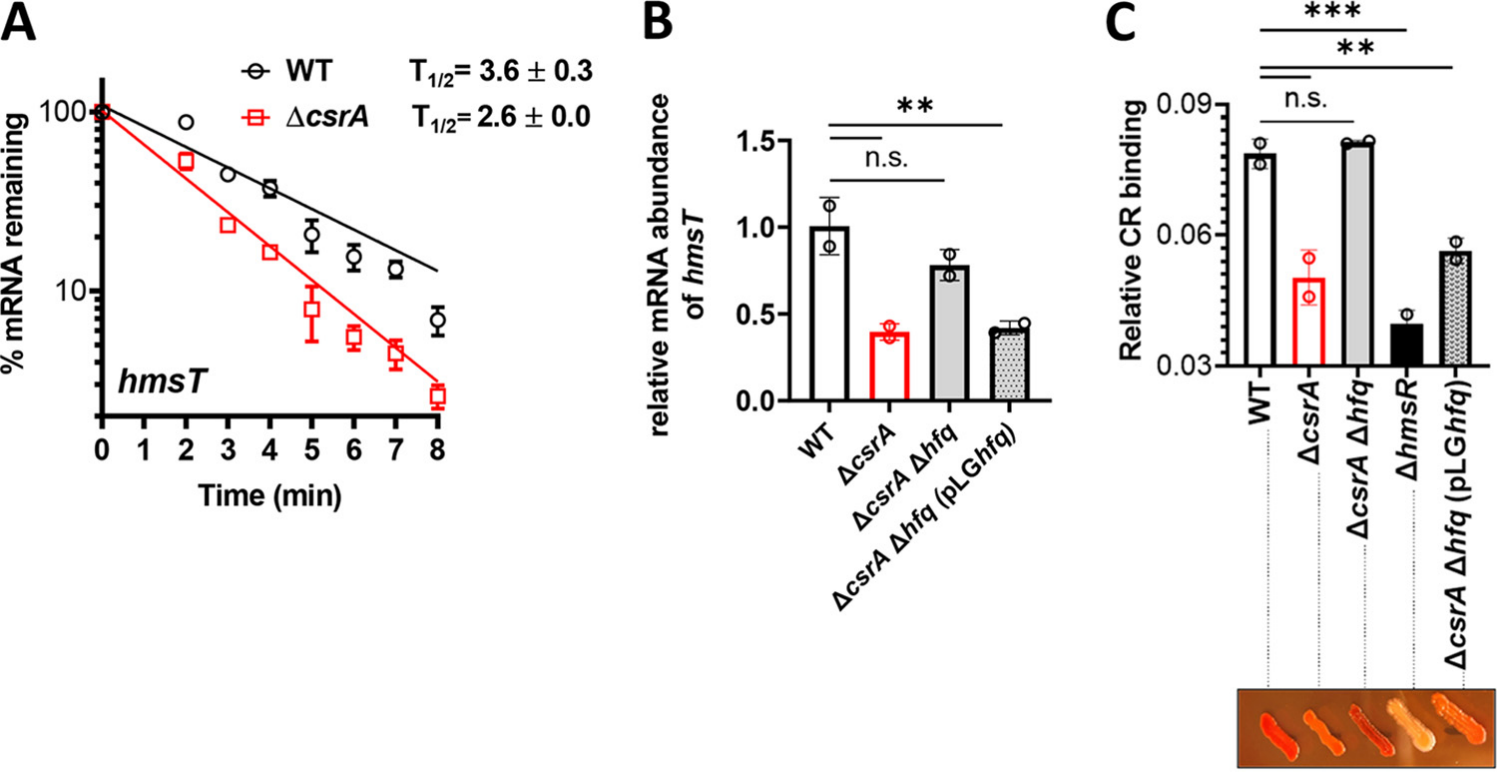
Epistasis analysis confirms that CsrA indirectly derepresses *hmsT* mRNA translation by targeting the *hfq* mRNA. (A) *hmsT* mRNA stability was determined as described for Fig. 5A. (B) Steady-state transcript levels of *hmsT* were compared between the WT (same as Fig. 2C), Δ*csrA* (same as Fig. 2C), Δ*csrA* Δ*hfq*, and complemented Δ*csrA* Δ*hfq* (pLG*hfq*) strains. Means ± SD from two independent experiments are shown. (C) Congo red (CR) binding assays were used to quantify EPS production in strains cultured in LB medium. A representative picture of strains grown for 48 h on LB containing CR is shown below. Error bars represent the mean ± SD bound CR from two independent experiments. Statistical significance was determined using one-way ANOVA with a Dunnett’s multiple-comparison posttest (**, *P* < 0.01; ***, *P* = 0.0005; n.s., not significant).

Y. pestis Hfq posttranscriptionally represses *hmsT* mRNA translation by accelerating its decay rate (22, 23). Therefore, the *hmsT* mRNA half-life should be shorter in a Δ*csrA* strain if CsrA no longer represses the *hfq* mRNA. To determine the mRNA stability of the *hmsT* mRNA in the Δ*csrA* strain versus the WT strain, we determined the half-life of the *hmsT* mRNA as described above. As predicted, the mean ± SD half-life of the *hmsT* mRNA (Fig. 6A) was reduced by 27.8% in the Δ*csrA* strain (2.6 ± 0.0 min) compared to the WT strain (3.6 ± 0.3 min).

We reasoned further that if CsrA represses *hfq* mRNA translation to facilitate derepression of *hmsT* mRNA translation, then a Δ*csrA* Δ*hfq* strain should show reestablishment of *hmsT* mRNA levels and increased biofilm production. To determine if this occurs, we generated a Δ*csrA* Δ*hfq* double mutant strain. A complemented derivative thereof, Δ*csrA* Δ*hfq* (pLG*hfq*) strain, was also constructed by inserting the *hfq* gene and native promoter sequence on a low-copy-number plasmid, pLG338, to create pLG*hfq*. First, steady-state levels of the *hmsT* mRNA at log phase were evaluated in the Δ*csrA* Δ*hfq* and Δ*csrA* Δ*hfq* (pLG*hfq*) strains compared to the Δ*csrA* and WT strains in TMH-gal (Fig. 6B). As hypothesized, the Δ*csrA* Δ*hfq* strain exhibited restored levels of the *hmsT* mRNA comparable to the WT strain. Additionally, the Δ*csrA* Δ*hfq* (pLG*hfq*) complemented strain showed *hmsT* levels comparable to the Δ*csrA* strain and significantly lower than that of the WT strain.

During routine lab culture, Y. pestis incurs spontaneous loss of a 102-kb locus, referred to as the pigmentation locus (56), or Pgm locus, named for the ability to form pigmented colonies on CR-supplemented agar (57). The *hmsHFRS* operon is located within the *pgm* locus and confers this phenotype. When culturing our Δ*csrA* Δ*hfq* strain on media that promote high biofilm production, we noted that the strain had a high propensity to form nonpigmented colonies, reflecting loss of EPS production. Therefore, to quantify EPS levels in these strains, CR assays were performed using LB medium that does not support high levels of biofilm production. Nonetheless, EPS levels in the Δ*csrA* Δ*hfq* strain were not different from those of the WT strain (Fig. 6C), and the Δ*csrA* Δ*hfq* (pLG*hfq*) complemented strain showed EPS levels comparable to those of the Δ*csrA* strain but significantly lower than that of the WT strain (Fig. 6C). Additionally, we analyzed the pigmentation phenotypes of the strains on LB agar supplemented with CR. Pigmentation phenotypes matched the quantitative CR binding assay data. Thus, as hypothesized, EPS and *hmsT* mRNA steady-state levels in the Δ*csrA* Δ*hfq* strain and Δ*csrA* Δ*hfq* (pLG*hfq*) complemented strain corresponded with that of the WT and Δ*csrA* strains, respectively.

### CsrA is required for the robust formation of biofilm-mediated blockage in fleas.

**FIG 7:**
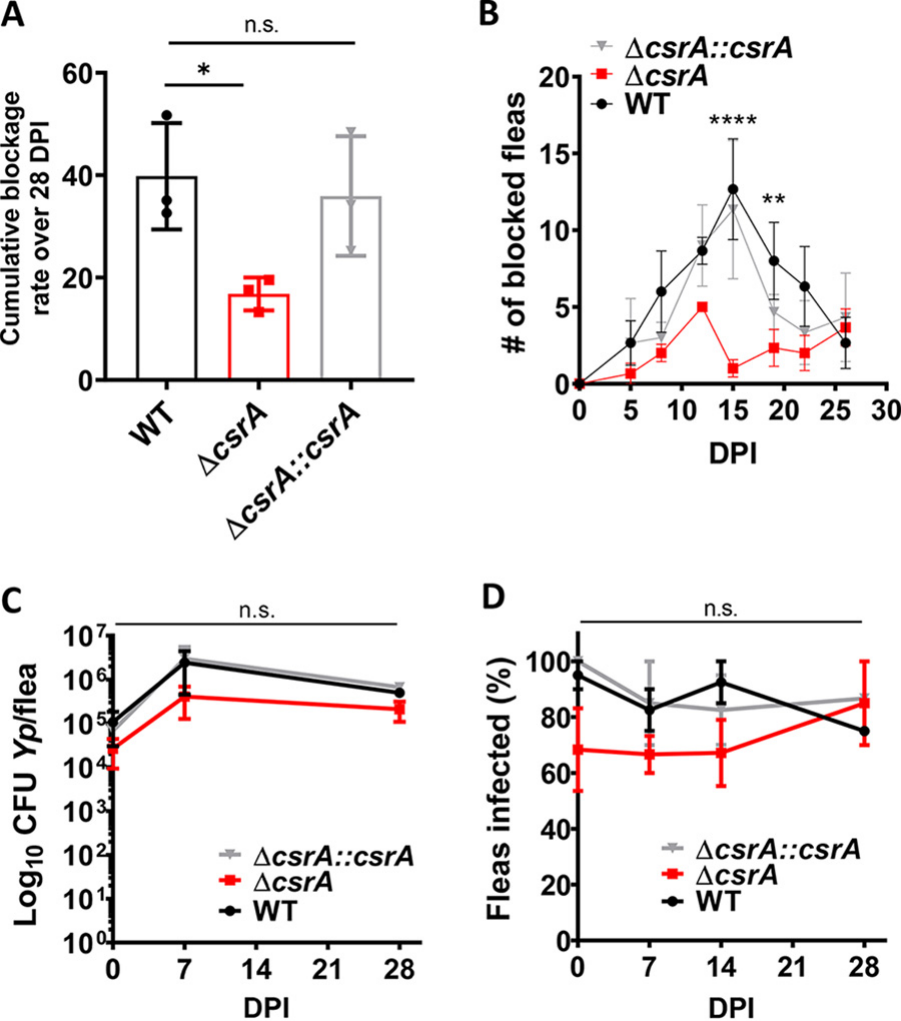
Flea foregut blockage and infection dynamics of the Δ*csrA* strain. Cohorts of Xenopsylla cheopis fleas were artificially infected with the WT (black), Δ*csrA* (red), or Δ*csrA*::*csrA* (gray) strains and monitored for blockage over 28 days postinfection (dpi). (A) Cumulative flea blockage rate of each strain is shown for 100 fleas (50 male, 50 female), with error bars representing the means ± SEM from three independent experiments. One-way ANOVA with Holm Sidak’s multiple-comparison posttest was used to determine statistical significance (*, *P* < 0.05; n.s., not significant). (B) Number of fleas blocked on 5, 8, 12, 15, 19, 22, and 26 dpi for each strain is shown as means ± SEM for three independent experiments. Two-way ANOVA with Holm Sidak’s multiple-comparison posttest was used to determine statistical significance for each time point (**, *P* < 0.01; ****, *P* < 0.0001). (C) Mean number of CFU per flea (*n* = 10 to 20) at 0, 7, and 28 dpi. Error bars represent means ± SEM from 2 to 3 independent experiments. Two-way ANOVA was used to test for statistical significance (n.s., not significant). (D) Percentage of fleas infected (*n* = 10 to 20) at 0, 7, and 28 dpi. Error bars represent means ± SEM from 2 to 3 independent experiments. Two-way ANOVA was used to test for statistical significance (n.s., not significant).

Finally, to define the role of CsrA in biofilm-mediated flea foregut blockage, we compared cumulative blockage rates in cohorts of rat fleas infected with WT, Δ*csrA*, and Δ*csrA*::*csrA* strains over a 28-day period (Fig. 7A). Fleas infected with the Δ*csrA* strain achieved significantly lower cumulative blockage rates of 16.8% (±SD, 3.2) relative to the WT strain-infected fleas, which achieved rates of 39.8% (±SD, 10.4). Similar to the WT strain-infected fleas, the Δ*csrA*::*csrA* strain-infected fleas exhibited rates of 34.2% (±SD, 11.7). An analysis of temporal incidence of blockage showed that while fleas infected with the WT and the Δ*csrA*::*csrA* strains attained peak blockage incidence at ∼15 days postinfection (dpi), the Δ*csrA* strain-infected fleas exhibited generally low blockage incidence, which was significant at 15 and 19 dpi (Fig. 7B). Flea bacterial loads and flea infection rates were not significantly different among the strains (Fig. 7C and D) despite being slightly lower in the Δ*csrA*-infected fleas. Therefore, growth kinetics of the Δ*csrA* strain in fleas was likely slightly lower, similar to that reported during *in vitro* growth (44). CsrA mutants in other bacterial species also show slow growth kinetics (28, 58). Additionally, the bacterial loads and infection rates of the Δ*csrA* strain resembled those reported for biofilm-deficient Y. pestis strains (7, 59–61). Biofilm is thought to maintain bacteria in aggregates that are not easily cleared through defecation after flea blood-feeding and digestion (reviewed in reference 3), accounting for lower bacterial number in strains with reduced biofilm levels.

## DISCUSSION

**FIG 8:**
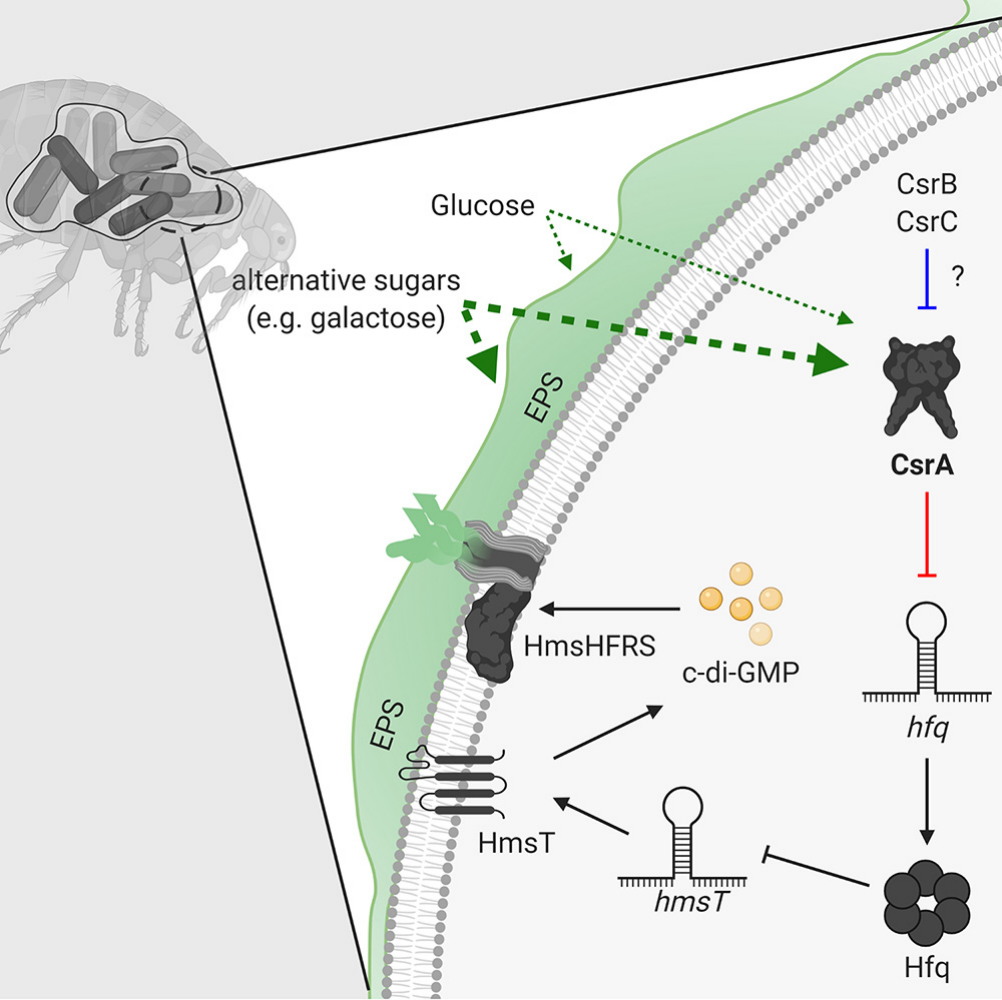
Current model for the CsrA-dependent regulation of c-di-GMP synthesis and biofilm formation in Y. pestis. In Y. pestis, the HmsHFRS proteins synthesize and export EPS to outside the cell to propagate biofilm formation, which leads to biofilm-mediated blockage in fleas. CsrA transduces alternative carbon metabolism cues to enhance biofilm EPS production (green thick broken arrow). CsrA does this through indirect positive regulation of the c-di-GMP synthesis enzyme, HmsT. Hfq represses *hmsT* mRNA translation by promoting its mRNA decay, but CsrA binds to two binding sites in the 5′UTR of *hfq* to inhibit its translation (solid red line), thereby alleviating repression of the *hmsT* mRNA translation by Hfq. How and under which physiological conditions ncRNAs CsrB and CsrC antagonize CsrA activity (solid blue line) in Y. pestis are yet to be determined.

Our work provides evidence for CsrA control of biofilm production in Y. pestis occurring through a tiered posttranscriptional regulatory mechanism (Fig. 8). In summary, cues from alternative carbon catabolism are transduced by CsrA to repress *hfq* mRNA translation, which, in turn, leads to translational derepression of the *hmsT* mRNA and a respective increase in intracellular c-di-GMP pools and biofilm production. Thus, CsrA is required to facilitate robust Y. pestis biofilm-mediated foregut blockage rates in rat fleas. Maintenance of normal blockage rates drives epizootic-scale plague transmission events (2, 62). Thus, an ∼50% reduction in blockage rate, as seen for the *csrA* mutant, is synonymous with a compromised ability to maintain natural plague transmission cycles. Coincidently, an *hmsT* mutant exhibits a similar ∼50% decrease in blockage rate (20, 21), in agreement with the notion that *csrA* mutant blockage rates are due to the absence of CsrA-dependent enhancement of *hmsT* mRNA translation.

Using ribose- and galactose-supplemented media enabled our reiteration that Y. pestis EPS levels are significantly greater during culture on alternative rather than primary carbon sources (44, 63). This aligns with observations that metabolic genes involved in uptake of alternative sugars, particularly pentose sugars, are strongly induced in blocked fleas (4, 5), and an intact pentose phosphate pathway is required for efficient flea foregut blockage (61). In addition, we demonstrated that severe impairment in EPS levels in the *csrA* mutant resulted from exacerbated defects in producing c-di-GMP on alternative carbon sources. CsrA is therefore critical for stimulating c-di-GMP synthesis to increase EPS levels coincident with alternative carbon metabolism. Improved growth and biofilm production on alternative carbon sources in Y. pestis is mediated by cyclic AMP (cAMP) and the cAMP receptor protein complex, cAMP-CRP, under glucose-limiting conditions (44, 63). Thus, Y. pestis CsrA-dependent biofilm formation overlaps carbon catabolite repression enabling biofilm production. Interestingly, in Y. pseudotuberculosis, cAMP-CRP activates *csrC* but represses *csrB* transcription, thereby optimizing infection fitness to the nutritional status of the mammalian host (64). CsrB also negatively regulates Y. pseudotuberculosis HmsHFRS-dependent biofilms (65). In E. coli, cAMP-CRP interfaces with the Csr system by inhibiting *csrC* and *csrB* expression and promoting CsrA-dependent biofilm inhibition (41, 66). If and how Y. pestis orthologs of sensory ncRNAs CsrB and CsrC (67) contribute to finely adjust CsrA activity in response to physiological changes during flea infection is under our investigation currently.

Bellows et al. (22) described that the *hmsT* mRNA is posttranscriptionally repressed by Hfq binding to its 5′ UTR, leading to accelerated transcript decay and decreases in transcript abundance of the *hmsT* mRNA. Hfq also posttranscriptionally regulates the *hmsT* mRNA through its AU-rich, long 3′ UTR sequence (23). A yet-to-be-identified small ncRNA is predicted to facilitate Hfq-*hmsT* mRNA interactions (22, 23). Consistent with these reports, we demonstrated that CsrA directly repressed *hfq* mRNA translation and that the *hmsT* mRNA incurred accelerated transcript decay. Epistatic analysis using a *csrA hfq* double mutant, in which we observed a restoration of *hmsT* mRNA and EPS levels to that exhibited by the wild-type strain, reinforced our findings. Notably, the *csrA hfq* double mutant exhibited slow growth kinetics relative to the other strains particularly when the Pgm locus was retained. However, when the Pgm locus was lost, the strain grew relatively faster (data not shown), suggesting that high biofilm production compromises growth fitness. Thus, CsrA in Y. pestis may play a role in optimizing growth fitness under nutritional conditions that promote costly biofilm production.

Hfq indirectly activates transcription of *hmsP*, and, together with posttranscriptional repression of the *hmsT* mRNA, causes decreases in biofilm and c-di-GMP levels in Y. pestis cultured on brain-heart infusion medium (22). However, in TMH-gal we noted that in an *hfq* mutant strain, the mRNA levels of *hmsT* were significantly greater than that of the wild-type strain, as expected, whereas *hmsP* transcript levels were similar between the wild-type and *hfq* mutant strains (see Fig. S3 in the supplemental material). We also showed that CsrA does not affect *hmsP* mRNA steady-state or translational levels, thereby eliminating a role for CsrA-dependent regulation of the *hmsP* mRNA in biofilm formation under biologically relevant conditions.

10.1128/mBio.01358-21.3 FIG S3 Steady-state mRNA levels of *hmsP*, *hmsT*, and *nlpD* in an *hfq* mutant. Steady-state gene expression levels of *hmsP* and *hmsT* were compared between the WT and Δ*hfq* strains grown to mid-log phase in TMH-gal. The housekeeping gene *nlpD* was used as a negative control. Data represent means from two independent experiments. An unpaired *t* test was used to test for statistical significance (*, *P* < 0.05; n.s., not significant). Download FIG S3, TIF file, 0.3 MB. Copyright © 2021 Silva-Rohwer et al. 2021 Silva-Rohwer et al. https://creativecommons.org/licenses/by/4.0/ This content is distributed under the terms of the Creative Commons Attribution 4.0 International license.

In E. coli MG1655, CsrA facilitates repression of an *hfq* mRNA originating from a promoter immediately upstream of the *hfq* ATG start through occlusion of ribosome binding and not accelerated mRNA decay (49). Coincidently, the CsrA binding site at the Shine-Dalgarno site for *hfq* is identical between Y. pestis and E. coli (Fig. S4). However, unlike Y. pestis, E. coli MG1655 does not similarly encode other GGA motifs upstream of the *hfq* mRNA translational start codon (Fig. S4). In Y. pestis, *hfq* mRNA transcribed with the upstream *miaA* gene fully restores Hfq function to an *hfq* mutant (50), emphasizing the significance of this transcript for production of functional Hfq levels. CsrA repression of this *miaA*-*hfq*-containing transcript in Y. pestis likely transpires through the two CsrA binding sites or the binding site at the Shine-Dalgarno site when the *hfq* transcript is derived from the immediately upstream promoter. In this way, *hfq* mRNA translation is expected to be more robustly inhibited by CsrA. Furthermore, the Y. pestis *hfq* transcript mutated only at the Shine-Dalgarno site possessed only a minor inability to completely shift (Fig. 3C). This may reflect an insufficiency of this binding site to properly repress *hfq* translation of the *miaA*-*hfq*-containing transcript, hence the need for the second binding site.

10.1128/mBio.01358-21.4 FIG S4 Alignment and mapping of GGA motifs located in the upstream sequences of *hfq* in E. coli and Y. pestis. Upstream sequences of E. coli (MG1655) and Y. pestis KIM6+. The transcriptional start sites are indicated. The GGA motifs are denoted BS1 and BS2, and the conserved CsrA binding site (BS1) located at the Shine-Dalgarno sequence in both Y. pestis and E. coli is underlined. Download FIG S4, TIF file, 1.1 MB. Copyright © 2021 Silva-Rohwer et al. 2021 Silva-Rohwer et al. https://creativecommons.org/licenses/by/4.0/ This content is distributed under the terms of the Creative Commons Attribution 4.0 International license.

In numerous pathogens, Hfq is critical for mammalian virulence (68, 69), and in Y. pestis it is also essential for flea foregut blockage (47). Hfq regulates distinct ncRNA repertoires required for physiological fitness that are conditionally expressed during changing infection stages (68, 69). The highly stable nature of the *hfq* mRNA and repression of *hfq* mRNA translation without mRNA decay revealed by our half-life studies conceivably preserves *hfq* mRNA levels for any future critical rapid redeployment of Hfq protein. CsrA-mediated alterations in the *hfq* mRNA chiefly occurred when the number of blocked rat fleas seen was at its peak. Whether, during this peak blockage stage, (i) the ncRNA that facilitates the Hfq-*hmsT* mRNA interaction is produced, requiring CsrA repression of the *hfq* mRNA and (ii) ncRNAs CsrB and CsrC are not expressed to prevent sequestration of CsrA are intriguing questions.

CsrA-dependent biofilm regulation can occur through multiple mechanisms in bacteria. For example, in E. coli, CsrA represses translation of mRNAs encoding DGCs (25), PgaABCD, a homolog to HmsHFRS (36), and the NhaR transcriptional regulator that activates *pgaABCD* transcription (70) but promotes translation of the *ymdA* mRNA involved in inhibiting biofilm production (54). Similarly, we predict that CsrA targets other mRNAs involved in biofilm formation in Y. pestis. One putative Y. pestis target that encodes a CsrA GGA binding motif in its 5′ UTR is the *hmsHFRS* mRNA (44). Future studies will be needed to validate putative mRNA targets in Y. pestis. Nonetheless, indirect modulation of DGC mRNAs to promote biofilm production, as illustrated here for CsrA in Y. pestis, is seldom appreciated as part of the repertoire of CsrA-mediated molecular mechanisms of biofilm control. Notably, this contrasts with the better-known paradigmatic role of CsrA direct translational repression of DGC mRNAs to inhibit biofilm formation in other Gammaproteobacteria.

## MATERIALS AND METHODS

### Bacterial strains, plasmids, and growth conditions.

Bacterial strains and plasmids used in this study are listed in Table S1 in the supplemental material. The Y. pestis KIM6+ (pCD1^−^) strains were cultured on Congo red-heart infusion agar (57) to confirm the presence of the *hmsHFRS* locus prior to subsequent culturing. Strains were grown at 25°C with shaking unless otherwise stated. The chemically defined TMH medium was prepared as described previously (46). DNA sequencing verified all constructs.

10.1128/mBio.01358-21.5 TABLE S1 Bacterial strains and plasmids used in this study. Download Table S1, DOCX file, 0.02 MB. Copyright © 2021 Silva-Rohwer et al. 2021 Silva-Rohwer et al. https://creativecommons.org/licenses/by/4.0/ This content is distributed under the terms of the Creative Commons Attribution 4.0 International license.

### Construction of Y. pestis mutant and complemented strains.

Primers are listed in Table S2 under “Mutant and complementation strain construction.” To generate the Y. pestis Δ*csrA*::*csrA* complemented *csrA* mutant strain, the *csrA* gene, with flanking promoter, and terminator regions were cloned into pUC18R6KT-mini-Tn7T-Km (71) at the EcoRI sites to create pUC18R6KT-mini-Tn7T-Km-*csrA*. This plasmid was then used to transpose the *csrA* expression fragment into the *glmS-pstS* site of the previously generated *csrA* mutant strain (44) as previously described (71).

10.1128/mBio.01358-21.6 TABLE S2 Oligonucleotides used in this study. Download Table S2, DOCX file, 0.02 MB. Copyright © 2021 Silva-Rohwer et al. 2021 Silva-Rohwer et al. https://creativecommons.org/licenses/by/4.0/ This content is distributed under the terms of the Creative Commons Attribution 4.0 International license.

To create the Δ*csrA* Δ*hfq* mutant strain, the *hfq* gene in the *csrA* mutant strain was replaced by a kanamycin resistance cassette by homologous recombination, as reported in a previous study (47). This was done by PCR amplification of a fragment containing flanking regions of the *hfq* gene and kanamycin cassette from the *hfq* mutant (47) genomic DNA using *hfq* deletion primers (Table S2). The *hfq* gene and its up- and downstream sequences were PCR amplified (Table S2) and cloned into a low-copy-number vector, pLG338 (72), at the SmaI site to create plasmid pLG*hfq*. The Δ*csrA* Δ*hfq* strain was transformed with pLG*hfq* to generate the Δ*csrA* Δ*hfq* (pLG*hfq*) complemented strain.

### CR binding assay.

CR binding assays were performed as previously described, with minor modifications (17). CR was used at final concentrations of 0.03 ng/ml (HIB), 0.06 ng/ml (TMH-glu), 0.12 ng/ml (TMH-gal or TMH-rib), or 0.02 ng/ml (LB) to account for EPS production and incubated for 3 h (HIB and LB) or 1 h (TMH). The *A*_500_ values of sample supernatants were subtracted from medium controls containing CR at their respective concentrations to calculate relative bound CR. The value for the WT strain under each condition was set to 1 for Fig. 1. These values then were multiplied by either 0.5, 1, or 4 for HIB, TMH-glu, and TMH-gal/rib to correct for the amount of CR added per medium condition.

### C-di-GMP extraction and quantification.

Strains were grown to late log phase. C-di-GMP was extracted from pelleted cells with extraction buffer (100 μl/48-mg cell pellet), and samples were neutralized and quantified using high-performance liquid chromatography (HPLC) as described previously (16, 18, 22).

### Fusion reporter construction and assay.

The upstream sequences and partial coding sequences were PCR amplified (primers are listed in Table S2 under “Translational fusion reporters”) for *flhDC*, *gyrB*, *hmsP*, *hmsT*, and *hfq*. The generated fragments were then fused to amplified fragments of *gfpmut3.1* from pFU34 (73) by splice overlap extension PCR (SOE-PCR) and then subjected to digestion with EcoRI and cloned into low-copy-number plasmid pMWO78 (74) at the EcoRI/SmaI sites. Plasmids generated were denoted pMWO78::*5’UTR of interest*-*gfpmut3.1* and transformed into the WT and Δ*csrA* strains (Table S1).

GFP reporter strains were grown to early log phase, split into separate flasks, and treated with anhydrotetracycline (ATc; 200 ng/ml) or vehicle. At 3 h postinduction, the numbers of relative fluorescent units (RFU; excitation, 475 nm; emission, 515 nm) were measured on a TECAN Spark plate reader with optical density at 600 nm (OD_600_) values taken simultaneously. The medium blank RFU reading was subtracted from the culture RFU of samples and normalized to OD_600_ values to account for bacterial growth. The difference of RFU/OD_600_ values of induced samples from uninduced samples then was calculated.

### CsrA-His_6_ expression and purification.

The *csrA* gene was amplified (primers listed in Table S2 under “CsrA-his tag construct”), digested with NcoI/XhoI, and cloned into matching sites in pET28A (Novagen) to generate pET28::*csrA-his*_6_, which was transformed into the E. coli strain, Bl21λDE3 pLysS. Cultures were induced as previously described (75). Cell pellets were resuspended in protein buffer (100 mM Tris‐HCl, 300 mM NaCl, pH 7.5, 20 mM imidazole), 26 U/ml Benzonase (Sigma), and one tablet of cOmplete Mini EDTA-free protease inhibitor cocktail (Roche) per 10 ml buffer and then lysed by sonication. Protein was purified from the supernatant using affinity chromatography as previously described (75). Relevant fractions were dialyzed with a 10,000 molecular weight cutoff (MWCO) Slide-A-Lyzer G2 dialysis cassette (Thermo Fisher Scientific) in dialysis buffer (protein buffer without imidazole). Buffer exchange was performed with 100 mM Tris-HCl, pH 7.5, using a Pall Microsep centrifugal device (3,000 MWCO). Concentrated protein was quantified using the DC protein assay (Bio-Rad). Before incubation with RNA probe, CsrA-His_6_ was prepared using 10 mM Tris‐HCl, 10% glycerol, pH 7.5. CsrA protein was diluted as previously described (76).

### Construction of *hfq* mRNA probes with GGA site mutations.

The nucleotide fragment containing a T7 promoter, the upstream region of *hfq* (bp −177 through 74), and the GG-to-CC mutation in BS1 was commercially synthesized (Eurofins) and blunt-end cloned into pJet1.2 (Thermo Fisher Scientific) to create pJet1.2::*hfq* UTR BS1mut. To generate the BS2 and BS1/BS2 fragments, primers (Table S2 under “EMSA probes”) containing the GGA-to-CCC mutation in BS2 or CC-to-GG restoration in BS1 were used with inverse PCR of the pJet1.2::*hfq* UTR BS1mut to generate mutated fragments for labeled probe generation.

### REMSA.

To generate biotin-labeled EMSA probes, PCR fragments were first generated from primers (Table S2 under “EMSA probes”) where each forward primer contained a T7 promoter sequence. Fragments were gel purified and transcribed with the MegaShortScript T7 transcription kit (Invitrogen). Probes were purified using the RNA Clean and Concentrator-25 kit (Zymo) and their size confirmed by electrophoresis. RNA probes were then labeled using a Pierce RNA 3′-end biotinylation kit and purified with the Oligo Clean and Concentrator kit (Zymo). Labeled RNA concentrations were determined using a Thermo Scientific NanoDrop.

CsrA-His_6_ probe binding reactions were conducted in 10× CsrA binding buffer (76) and incubated at 37°C for 30 min. For REMSAs shown in Fig. 4, binding reaction mixtures included 60 ng unlabeled yeast RNA (Invitrogen) and SUPERase-In (Invitrogen). CsrA-His_6_–probe complexes were electrophoresed on a 6% nondenaturing polyacrylamide gel, transblotted to a positively charged nylon membrane, and then UV cross-linked (120 mJ/cm^2^). Biotinylated probes were detected using the chemiluminescent nucleic acid detection module (Thermo Fisher Scientific).

### *In vitro* cell-free translation assay.

The PURExpress kit (New England Biolabs) was used per the manufacturer’s instructions. The reporter fusion constructs pMWO78::*hfq*-*gfpmut3.*1 or pMWO78::*hmsT*-*gfpmut3.*1, described above, were used as templates to generate *hfq-gfp* and *hmsT-gfp* mRNA transcripts with primers containing a T7 promoter sequence (Table S2 under “EMSA probes”) using the MegaShortScript T7 transcription kit. mRNA transcripts were purified with the RNA Clean and Concentrator-25 kit (Zymo). Reaction mixtures contained 326 nM mRNA transcript with 6.2 μM CsrA-His_6_ and were incubated at 37°C. GFP signal was detected by immunoblot using 1:20,000 rabbit anti-GFP (Invitrogen), 1:100,000 goat anti-rabbit horseradish peroxidase (Invitrogen), and the SuperSignal West Femto kit (Thermo Fisher Scientific) and quantified by densitometry on a ChemicDoc MP using Image Lab 4.1.

### Quantification of steady-state mRNA levels and mRNA stability assays.

Samples were added to RNAprotect bacterial reagent (Qiagen). RNA isolation, qRT-PCR (Table S2 under “RT-qPCR”), and 2^−ΔΔ^*^CT^* analysis was conducted as previously described (77). For steady-state mRNA analysis, samples were collected from strains grown to log phase. For mRNA stability assays, rifampin (400 μg/ml) was added to strains grown to log phase. To quantify *hfq* mRNA half-life, samples were collected at 0 (prerifampin) and 3, 6, 9, 12, and 15 min postrifampin. To quantify *hmsT* mRNA half-life, samples were collected at 0 (prerifampin) and 2, 3, 4, 5, 6, 7, and 8 min postrifampin addition. Percent mRNA remaining relative to *t* = 0 (set to 100%) was plotted on semilog graphs.

### Flea infections.

Cohorts of Xenopsylla cheopis fleas were artificially infected with Y. pestis strains, and infected flea maintenance, blockage, and CFU enumeration were performed as previously described (21, 78). To account for growth defects in the Δ*csrA* strain, strains were grown overnight at room temperature and then moved to 37°C at 5 h prior to infection. Studies with mice were performed in strict accordance with the U.S. National Institutes of Health (NIH) *Guide for the Care and Use of Laboratory Animals* (79) and as approved by the Washington State University Institutional Animal Care and Use Committee.

### Statistical analysis.

Details of statistical analysis using GraphPad Prism version 8.1.1 are provided in the legends of Fig. 1 to 7.

## References

[1] Perry RD, Fetherston JD. 1997. *Yersinia pestis*–etiologic agent of plague. Clin Microbiol Rev 10:35–66. doi:10.1128/CMR.10.1.35. 8993858 PMC172914

[2] Hinnebusch BJ, Bland DM, Bosio CF, Jarrett CO. 2017. Comparative ability of *Oropsylla montana* and *Xenopsylla cheopis* fleas to transmit *Yersinia pestis* by two different mechanisms. PLoS Negl Trop Dis 11:e0005276. doi:10.1371/journal.pntd.0005276. 28081130 PMC5230758

[3] Hinnebusch BJ, Jarrett CO, Bland DM. 2017. Fleaing the plague: adaptations of *Yersinia pestis* to its insect vector that lead to transmission. Annu Rev Microbiol 71:215–232. doi:10.1146/annurev-micro-090816-093521. 28886687

[4] Vadyvaloo V, Jarrett C, Sturdevant DE, Sebbane F, Hinnebusch BJ. 2010. Transit through the flea vector induces a pretransmission innate immunity resistance phenotype in *Yersinia pestis*. PLoS Pathog 6:e1000783. doi:10.1371/journal.ppat.1000783. 20195507 PMC2829055

[5] Vadyvaloo V, Jarrett C, Sturdevant D, Sebbane F, Hinnebusch BJ. 2007. Analysis of *Yersinia pestis* gene expression in the flea vector. Adv Exp Med Biol 603:192–200. doi:10.1007/978-0-387-72124-8_16. 17966415

[6] Bacot AW, Martin SCJ. 1914. Observations on the mechanism of the transmission of plague by fleas. J Hyg 13:423–439. PMC2167459 20474555

[7] Hinnebusch BJ, Perry RD, Schwan TG. 1996. Role of the *Yersinia pestis* hemin storage (*hms*) locus in the transmission of plague by fleas. Science 273:367–370. doi:10.1126/science.273.5273.367. 8662526

[8] Perry RD, Pendrak ML, Schuetze P. 1990. Identification and cloning of a hemin storage locus involved in the pigmentation phenotype of *Yersinia pestis*. J Bacteriol 172:5929–5937. doi:10.1128/jb.172.10.5929-5937.1990. 2211518 PMC526914

[9] Perry RD, Bobrov AG. 2010. Role of cyclic di-GMP in biofilm development and signaling in *Yersinia pestis*, p 270–281. *In* Wolfe AJ, Visick KL (ed), The second messenger cyclic di-GMP. ASM Press, Washington, DC. doi:10.1128/9781555816667.

[10] Perry RD, Bobrov AG, Kirillina O, Jones HA, Pedersen L, Abney J, Fetherston JD. 2004. Temperature regulation of the hemin storage (Hms+) phenotype of *Yersinia pestis* is posttranscriptional. J Bacteriol 186:1638–1647. doi:10.1128/JB.186.6.1638-1647.2004. 14996794 PMC355957

[11] Jenal U, Reinders A, Lori C. 2017. Cyclic di-GMP: second messenger extraordinaire. Nat Rev Microbiol 15:271–284. doi:10.1038/nrmicro.2016.190. 28163311

[12] Ryan RP. 2013. Cyclic di-GMP signalling and the regulation of bacterial virulence. Microbiology 159:1286–1297. doi:10.1099/mic.0.068189-0. 23704785 PMC3749722

[13] Valentini M, Filloux A. 2016. Biofilms and cyclic di-GMP (c-di-GMP) signaling: lessons from *Pseudomonas aeruginosa* and other bacteria. J Biol Chem 291:12547–12555. doi:10.1074/jbc.R115.711507. 27129226 PMC4933438

[14] Sun YC, Jarrett CO, Bosio CF, Hinnebusch BJ. 2014. Retracing the evolutionary path that led to flea-borne transmission of *Yersinia pestis*. Cell Host Microbe 15:578–586. doi:10.1016/j.chom.2014.04.003. 24832452 PMC4084870

[15] Hinnebusch BJ, Chouikha I, Sun YC. 2016. Ecological opportunity, evolution, and the emergence of flea-borne plague. Infect Immun 84:1932–1940. doi:10.1128/IAI.00188-16. 27160296 PMC4936347

[16] Bobrov AG, Kirillina O, Vadyvaloo V, Koestler BJ, Hinz AK, Mack D, Waters CM, Perry RD. 2015. The *Yersinia pestis* HmsCDE regulatory system is essential for blockage of the oriental rat flea (*Xenopsylla cheopis*), a classic plague vector. Environ Microbiol 17:947–959. doi:10.1111/1462-2920.12419. 25586342 PMC4295937

[17] Kirillina O, Fetherston JD, Bobrov AG, Abney J, Perry RD. 2004. HmsP, a putative phosphodiesterase, and HmsT, a putative diguanylate cyclase, control Hms-dependent biofilm formation in *Yersinia pestis*. Mol Microbiol 54:75–88. doi:10.1111/j.1365-2958.2004.04253.x. 15458406

[18] Bobrov AG, Kirillina O, Ryjenkov DA, Waters CM, Price PA, Fetherston JD, Mack D, Goldman WE, Gomelsky M, Perry RD. 2011. Systematic analysis of cyclic di-GMP signalling enzymes and their role in biofilm formation and virulence in *Yersinia pestis*. Mol Microbiol 79:533–551. doi:10.1111/j.1365-2958.2010.07470.x. 21219468 PMC3058942

[19] Ren GX, Yan HQ, Zhu H, Guo XP, Sun YC. 2014. HmsC, a periplasmic protein, controls biofilm formation via repression of HmsD, a diguanylate cyclase in *Yersinia pestis*. Environ Microbiol 16:1202–1216. doi:10.1111/1462-2920.12323. 24192006

[20] Sun YC, Koumoutsi A, Jarrett C, Lawrence K, Gherardini FC, Darby C, Hinnebusch BJ. 2011. Differential control of *Yersinia pestis* biofilm formation *in vitro* and in the flea vector by two c-di-GMP diguanylate cyclases. PLoS One 6:e19267. doi:10.1371/journal.pone.0019267. 21559445 PMC3084805

[21] Lemon A, Sagawa J, Gravelle K, Vadyvaloo V. 2020. Biovar-related differences apparent in the flea foregut colonization phenotype of distinct *Yersinia pestis* strains do not impact transmission efficiency. Parasit Vectors 13:335. doi:10.1186/s13071-020-04207-x. 32611387 PMC7329463

[22] Bellows LE, Koestler BJ, Karaba SM, Waters CM, Lathem WW. 2012. Hfq-dependent, co-ordinate control of cyclic diguanylate synthesis and catabolism in the plague pathogen *Yersinia pestis*. Mol Microbiol 86:661–674. doi:10.1111/mmi.12011. 22924957 PMC3480973

[23] Zhu H, Mao XJ, Guo XP, Sun YC. 2016. The *hmsT* 3' untranslated region mediates c-di-GMP metabolism and biofilm formation in *Yersinia pestis*. Mol Microbiol 99:1167–1178. doi:10.1111/mmi.13301. 26711808

[24] Sun YC, Guo XP, Hinnebusch BJ, Darby C. 2012. The *Yersinia pestis* Rcs phosphorelay inhibits biofilm formation by repressing transcription of the diguanylate cyclase gene *hmsT*. J Bacteriol 194:2020–2026. doi:10.1128/JB.06243-11. 22328676 PMC3318482

[25] Jonas K, Edwards AN, Simm R, Romeo T, Römling U, Melefors Ö. 2008. The RNA binding protein CsrA controls cyclic di-GMP metabolism by directly regulating the expression of GGDEF proteins: CsrA regulates GGDEF proteins. Mol Microbiol 70:236–257. doi:10.1111/j.1365-2958.2008.06411.x. 18713317 PMC2735045

[26] Jackson DW, Suzuki K, Oakford L, Simecka JW, Hart ME, Romeo T. 2002. Biofilm formation and dispersal under the influence of the global regulator CsrA of *Escherichia coli*. J Bacteriol 184:290–301. doi:10.1128/JB.184.1.290-301.2002. 11741870 PMC134780

[27] Dubey AK, Baker CS, Suzuki K, Jones AD, Pandit P, Romeo T, Babitzke P. 2003. CsrA regulates translation of the Escherichia coli carbon starvation gene, cstA, by blocking ribosome access to the cstA transcript. J Bacteriol 185:4450–4460. doi:10.1128/JB.185.15.4450-4460.2003. 12867454 PMC165747

[28] Lawhon SD, Frye JG, Suyemoto M, Porwollik S, McClelland M, Altier C. 2003. Global regulation by CsrA in *Salmonella typhimurium*. Mol Microbiol 48:1633–1645. doi:10.1046/j.1365-2958.2003.03535.x. 12791144

[29] LeGrand K, Petersen S, Zheng Y, Liu KK, Ozturk G, Chen J-Y, Young GM. 2015. CsrA impacts survival of *Yersinia enterocolitica* by affecting a myriad of physiological activities. BMC Microbiol 15:31. doi:10.1186/s12866-015-0343-6. 25885058 PMC4336687

[30] Romeo T, Babitzke P. 2018. Global regulation by CsrA and its RNA antagonists. Microbiol Spectr 6:10.1128/microbiolspec.RWR-0009-2017. doi:10.1128/microbiolspec.RWR-0009-2017. PMC5868435 29573256

[31] Irie Y, Starkey M, Edwards AN, Wozniak DJ, Romeo T, Parsek MR. 2010. *Pseudomonas aeruginosa* biofilm matrix polysaccharide Psl is regulated transcriptionally by RpoS and post-transcriptionally by RsmA. Mol Microbiol 78:158–172. doi:10.1111/j.1365-2958.2010.07320.x. 20735777 PMC2984543

[32] Fields JA, Thompson SA. 2012. *Campylobacter jejuni* CsrA complements an *Escherichia coli csrA* mutation for the regulation of biofilm formation, motility and cellular morphology but not glycogen accumulation. BMC Microbiol 12:233. doi:10.1186/1471-2180-12-233. 23051923 PMC3534301

[33] Lee JH, Ancona V, Chatnaparat T, Yang H-W, Zhao Y. 2019. The RNA-binding protein CsrA controls virulence in *Erwinia amylovora* by regulating RelA, RcsB, and FlhD at the posttranscriptional level. MPMI 32:1448–1459. doi:10.1094/MPMI-03-19-0077-R. 31140921

[34] Liu MY, Yang H, Romeo T. 1995. The product of the pleiotropic *Escherichia coli gene csrA* modulates glycogen biosynthesis via effects on mRNA stability. J Bacteriol 177:2663–2672. doi:10.1128/jb.177.10.2663-2672.1995. 7751274 PMC176935

[35] Lu X-H, An S-Q, Tang D-J, McCarthy Y, Tang J-L, Dow JM, Ryan RP. 2012. RsmA regulates biofilm formation in *Xanthomonas campestris* through a regulatory network involving cyclic di-GMP and the Clp transcription factor. PLoS One 7:e52646. doi:10.1371/journal.pone.0052646. 23285129 PMC3528676

[36] Wang X, Dubey AK, Suzuki K, Baker CS, Babitzke P, Romeo T. 2005. CsrA post-transcriptionally represses *pgaABCD*, responsible for synthesis of a biofilm polysaccharide adhesin of *Escherichia coli*. Mol Microbiol 56:1648–1663. doi:10.1111/j.1365-2958.2005.04648.x. 15916613

[37] Kulkarni PR, Jia T, Kuehne SA, Kerkering TM, Morris ER, Searle MS, Heeb S, Rao J, Kulkarni RV. 2014. A sequence-based approach for prediction of CsrA/RsmA targets in bacteria with experimental validation in *Pseudomonas aeruginosa*. Nucleic Acids Res 42:6811–6825. doi:10.1093/nar/gku309. 24782516 PMC4066749

[38] Leistra AN, Gelderman G, Sowa SW, Moon-Walker A, Salis HM, Contreras LM. 2018. A canonical biophysical model of the CsrA global regulator suggests flexible regulator-target interactions. Sci Rep 8:9892. doi:10.1038/s41598-018-27474-2. 29967470 PMC6028588

[39] Mercante J, Edwards AN, Dubey AK, Babitzke P, Romeo T. 2009. Molecular geometry of CsrA (RsmA) binding to RNA and its implications for regulated expression. J Mol Biol 392:511–528. doi:10.1016/j.jmb.2009.07.034. 19619561 PMC2735826

[40] Dubey AK, Baker CS, Romeo T, Babitzke P. 2005. RNA sequence and secondary structure participate in high-affinity CsrA-RNA interaction. RNA 11:1579–1587. doi:10.1261/rna.2990205. 16131593 PMC1370842

[41] Potts AH, Vakulskas CA, Pannuri A, Yakhnin H, Babitzke P, Romeo T. 2017. Global role of the bacterial post-transcriptional regulator CsrA revealed by integrated transcriptomics. Nat Commun 8:1596. doi:10.1038/s41467-017-01613-1. 29150605 PMC5694010

[42] Jonas K, Edwards AN, Ahmad I, Romeo T, Römling U, Melefors O. 2010. Complex regulatory network encompassing the Csr, c-di-GMP and motility systems of *Salmonella Typhimurium*. Environ Microbiol 12:524–540. doi:10.1111/j.1462-2920.2009.02097.x. 19919539 PMC2888478

[43] Huertas-Rosales Ó, Romero M, Heeb S, Espinosa-Urgel M, Cámara M, Ramos-González MI. 2017. The Pseudomonas putida CsrA/RsmA homologues negatively affect c-di-GMP pools and biofilm formation through the GGDEF/EAL response regulator CfcR. Environ Microbiol 19:3551–3566. doi:10.1111/1462-2920.13848. 28677348 PMC6849547

[44] Willias SP, Chauhan S, Lo C-C, Chain PSG, Motin VL. 2015. CRP-mediated carbon catabolite regulation of *Yersinia pestis* biofilm formation is enhanced by the carbon storage regulator protein, CsrA. PLoS One 10:e0135481. doi:10.1371/journal.pone.0135481. 26305456 PMC4549057

[45] Wood PJ. 1980. Specificity in the interaction of direct dyes with polysaccharides. Carbohydrate Res 85:271–287. doi:10.1016/S0008-6215(00)84676-5.

[46] Straley SC, Bowmer WS. 1986. Virulence genes regulated at the transcriptional level by Ca2+ in *Yersinia pestis* include structural genes for outer membrane proteins. Infect Immun 51:445–454. doi:10.1128/iai.51.2.445-454.1986. 3002984 PMC262351

[47] Rempe KA, Hinz AK, Vadyvaloo V. 2012. Hfq regulates biofilm gut blockage that facilitates flea-borne transmission of *Yersinia pestis*. J Bacteriol 194:2036–2040. doi:10.1128/JB.06568-11. 22328669 PMC3318476

[48] Heroven AK, Bohme K, Rohde M, Dersch P. 2008. A Csr-type regulatory system, including small non-coding RNAs, regulates the global virulence regulator RovA of *Yersinia pseudotuberculosis* through RovM. Mol Microbiol 68:1179–1195. doi:10.1111/j.1365-2958.2008.06218.x. 18430141

[49] Baker CS, Eöry LA, Yakhnin H, Mercante J, Romeo T, Babitzke P. 2007. CsrA inhibits translation initiation of *Escherichia coli hfq* by binding to a single site overlapping the Shine-Dalgarno sequence. J Bacteriol 189:5472–5481. doi:10.1128/JB.00529-07. 17526692 PMC1951803

[50] Bai G, Golubov A, Smith EA, McDonough KA. 2010. The importance of the small RNA chaperone Hfq for growth of epidemic *Yersinia pestis*, but not *Yersinia pseudotuberculosis*, with implications for plague biology. J Bacteriol 192:4239–4245. doi:10.1128/JB.00504-10. 20543069 PMC2916426

[51] Huang Y-H, Ferrières L, Clarke DJ. 2006. The role of the Rcs phosphorelay in *Enterobacteriaceae*. Res Microbiol 157:206–212. doi:10.1016/j.resmic.2005.11.005. 16427772

[52] Dugar G, Svensson SL, Bischler T, Wäldchen S, Reinhardt R, Sauer M, Sharma CM. 2016. The CsrA-FliW network controls polar localization of the dual-function flagellin mRNA in *Campylobacter jejuni*. Nat Commun 7:11667. doi:10.1038/ncomms11667. 27229370 PMC4894983

[53] Park H, McGibbon LC, Potts AH, Yakhnin H, Romeo T, Babitzke P. 2017. Translational repression of the RpoS antiadapter IraD by CsrA is mediated via translational coupling to a short upstream open reading frame. mBio 8:e01355-17. doi:10.1128/mBio.01355-17. 28851853 PMC5574718

[54] Renda A, Poly S, Lai YJ, Pannuri A, Yakhnin H, Potts AH, Bevilacqua PC, Romeo T, Babitzke P. 2020. CsrA-mediated translational activation of *ymdA* expression in *Escherichia coli*. mBio 11:e00849-20. doi:10.1128/mBio.00849-20. 32934077 PMC7492729

[55] Pourciau C, Lai Y-J, Gorelik M, Babitzke P, Romeo T. 2020. Diverse mechanisms and circuitry for global regulation by the RNA-binding protein CsrA. Front Microbiol 11:601352. doi:10.3389/fmicb.2020.601352. 33193284 PMC7652899

[56] Fetherston JD, Schuetze P, Perry RD. 1992. Loss of the pigmentation phenotype in *Yersinia pestis* is due to the spontaneous deletion of 102 kb of chromosomal DNA which is flanked by a repetitive element. Mol Microbiol 6:2693–2704. doi:10.1111/j.1365-2958.1992.tb01446.x. 1447977

[57] Surgalla MJ, Beesley ED. 1969. Congo red-agar plating medium for detecting pigmentation in *Pasteurella pestis*. Appl Microbiol 18:834–837. doi:10.1128/am.18.5.834-837.1969. 5370459 PMC378096

[58] Timmermans J, Van Melderen L. 2009. Conditional essentiality of the *csrA* gene in *Escherichia coli*. J Bacteriol 191:1722–1724. doi:10.1128/JB.01573-08. 19103924 PMC2648183

[59] Bouvenot T, Dewitte A, Bennaceur N, Pradel E, Pierre F, Bontemps-Gallo S, Sebbane F. 2021. Interplay between *Yersinia pestis* and its flea vector in lipoate metabolism. ISME J 15:1136–1149. doi:10.1038/s41396-020-00839-0. 33479491 PMC8182812

[60] Hinnebusch BJ. 2012. Biofilm-dependent and biofilm-independent mechanisms of transmission of *Yersinia pestis* by fleas. Adv Exp Med Biol 954:237–243. doi:10.1007/978-1-4614-3561-7_30. 22782769

[61] Dewitte A, Bouvenot T, Pierre F, Ricard I, Pradel E, Barois N, Hujeux A, Bontemps-Gallo S, Sebbane F. 2020. A refined model of how *Yersinia pestis* produces a transmissible infection in its flea vector. PLoS Pathog 16:e1008440. doi:10.1371/journal.ppat.1008440. 32294143 PMC7185726

[62] Lorange EA, Race BL, Sebbane F, Hinnebusch BJ. 2005. Poor vector competence of fleas and the evolution of hypervirulence in *Yersinia pestis*. J Infect Dis 191:1907–1912. doi:10.1086/429931. 15871125

[63] Ritzert JT, Minasov G, Embry R, Schipma MJ, Satchell KJF. 2019. The cyclic AMP receptor protein regulates quorum sensing and global gene expression in *Yersinia pestis* during planktonic growth and growth in biofilms. mBio 10:e02613-19. doi:10.1128/mBio.02613-19. 31744922 PMC6867900

[64] Heroven AK, Sest M, Pisano F, Scheb-Wetzel M, Steinmann R, Bohme K, Klein J, Munch R, Schomburg D, Dersch P. 2012. Crp induces switching of the CsrB and CsrC RNAs in *Yersinia pseudotuberculosis* and links nutritional status to virulence. Front Cell Infect Microbiol 2:158. doi:10.3389/fcimb.2012.00158. 23251905 PMC3523269

[65] Schachterle JK, Stewart RM, Schachterle MB, Calder JT, Kang H, Prince JT, Erickson DL. 2018. Yersinia pseudotuberculosis BarA-UvrY two-component regulatory system represses biofilms via CsrB. Front Cell Infect Microbiol 8:323. doi:10.3389/fcimb.2018.00323. 30280093 PMC6153318

[66] Pannuri A, Vakulskas CA, Zere T, McGibbon LC, Edwards AN, Georgellis D, Babitzke P, Romeo T. 2016. Circuitry linking the catabolite repression and Csr global regulatory systems of *Escherichia coli*. J Bacteriol 198:3000–3015. doi:10.1128/JB.00454-16. 27551019 PMC5055604

[67] Beauregard A, Smith EA, Petrone BL, Singh N, Karch C, McDonough KA, Wade JT. 2013. Identification and characterization of small RNAs in *Yersinia pestis*. RNA Biol 10:397–405. doi:10.4161/rna.23590. 23324607 PMC3672283

[68] Koo JT, Alleyne TM, Schiano CA, Jafari N, Lathem WW. 2011. Global discovery of small RNAs in *Yersinia pseudotuberculosis i*dentifies *Yersinia*-specific small, noncoding RNAs required for virulence. Proc Natl Acad Sci U S A 108:E709–E717. doi:10.1073/pnas.1101655108. 21876162 PMC3174644

[69] Schiano CA, Koo JT, Schipma MJ, Caulfield AJ, Jafari N, Lathem WW. 2014. Genome-wide analysis of small RNAs expressed by *Yersinia pestis* identifies a regulator of the Yop-Ysc type III secretion system. J Bacteriol 196:1659–1670. doi:10.1128/JB.01456-13. 24532772 PMC3993326

[70] Pannuri A, Yakhnin H, Vakulskas CA, Edwards AN, Babitzke P, Romeo T. 2012. Translational repression of NhaR, a novel pathway for multi-tier regulation of biofilm circuitry by CsrA. J Bacteriol 194:79–89. doi:10.1128/JB.06209-11. 22037401 PMC3256615

[71] Choi KH, Gaynor JB, White KG, Lopez C, Bosio CM, Karkhoff-Schweizer RR, Schweizer HP. 2005. A Tn7-based broad-range bacterial cloning and expression system. Nat Methods 2:443–448. doi:10.1038/nmeth765. 15908923

[72] Stoker NG, Fairweather NF, Spratt BG. 1982. Versatile low-copy-number plasmid vectors for cloning in *Escherichia coli*. Gene 18:335–341. doi:10.1016/0378-1119(82)90172-x. 6290337

[73] Uliczka F, Pisano F, Kochut A, Opitz W, Herbst K, Stolz T, Dersch P. 2011. Monitoring of gene expression in bacteria during infections using an adaptable set of bioluminescent, fluorescent and colorigenic fusion vectors. PLoS One 6:e20425. doi:10.1371/journal.pone.0020425. 21673990 PMC3108616

[74] Obrist MW, Miller VL. 2012. Low copy expression vectors for use in *Yersinia* sp. and related organisms. Plasmid 68:33–42. doi:10.1016/j.plasmid.2012.02.003. 22445322 PMC3367051

[75] Kusmierek M, Heroven AK, Beckstette M, Nuss AM, Dersch P. 2019. Discovering *Yersinia*-host interactions by tissue dual RNA-Seq. Methods Mol Biol 2010:99–116. doi:10.1007/978-1-4939-9541-7_8. 31177434

[76] Yakhnin AV, Yakhnin H, Babitzke P. 2012. Gel mobility shift assays to detect protein-RNA interactions. Methods Mol Biol 905:201–211. doi:10.1007/978-1-61779-949-5_12. 22736005 PMC4687016

[77] Martinez-Chavarria LC, Sagawa J, Irons J, Hinz AK, Lemon A, Graca T, Downs DM, Vadyvaloo V. 2020. Putative horizontally acquired genes, highly transcribed during *Yersinia pestis* flea infection, are induced by hyperosmotic stress and function in aromatic amino acid metabolism. J Bacteriol 202:e00733-19. doi:10.1128/JB.00733-19. 32205462 PMC7221256

[78] Lemon A, Cherzan N, Vadyvaloo V. 2020. Influence of temperature on development of *Yersinia pestis* foregut blockage in *Xenopsylla cheopis* (Siphonaptera: Pulicidae) and *Oropsylla montana* (Siphonaptera: Ceratophyllidae). J Med Entomol doi:10.1093/jme/tjaa113. 32533162

[79] National Research Council. 2011. Guide for the care and use of laboratory animals, 8th ed. National Academies Press, Washington, DC.

