# Supplementary figures and images for: CsrA Enhances Cyclic-di-GMP Biosynthesis and Yersinia pestis Biofilm Blockage of the Flea Foregut by Alleviating Hfq-Dependent Repression of the hmsT mRNA
Source: mBio. 2021 Aug 3;12(4):e01358-21. doi: 10.1128/mBio.01358-21 (PMC8406273; doi:10.1128/mBio.01358-21)

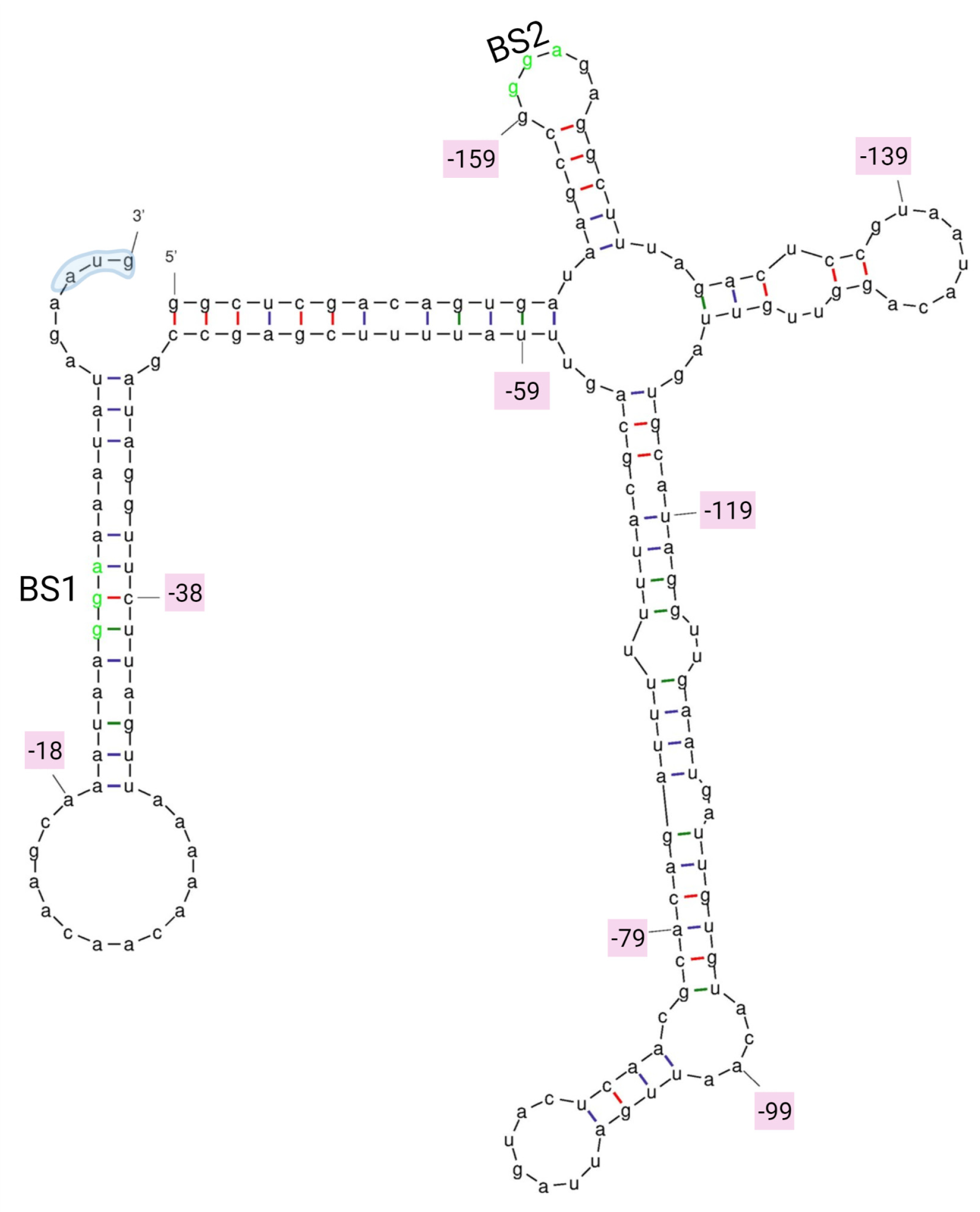

Supplement: FIG S1 [file mbio.01358-21-sf001.tif]

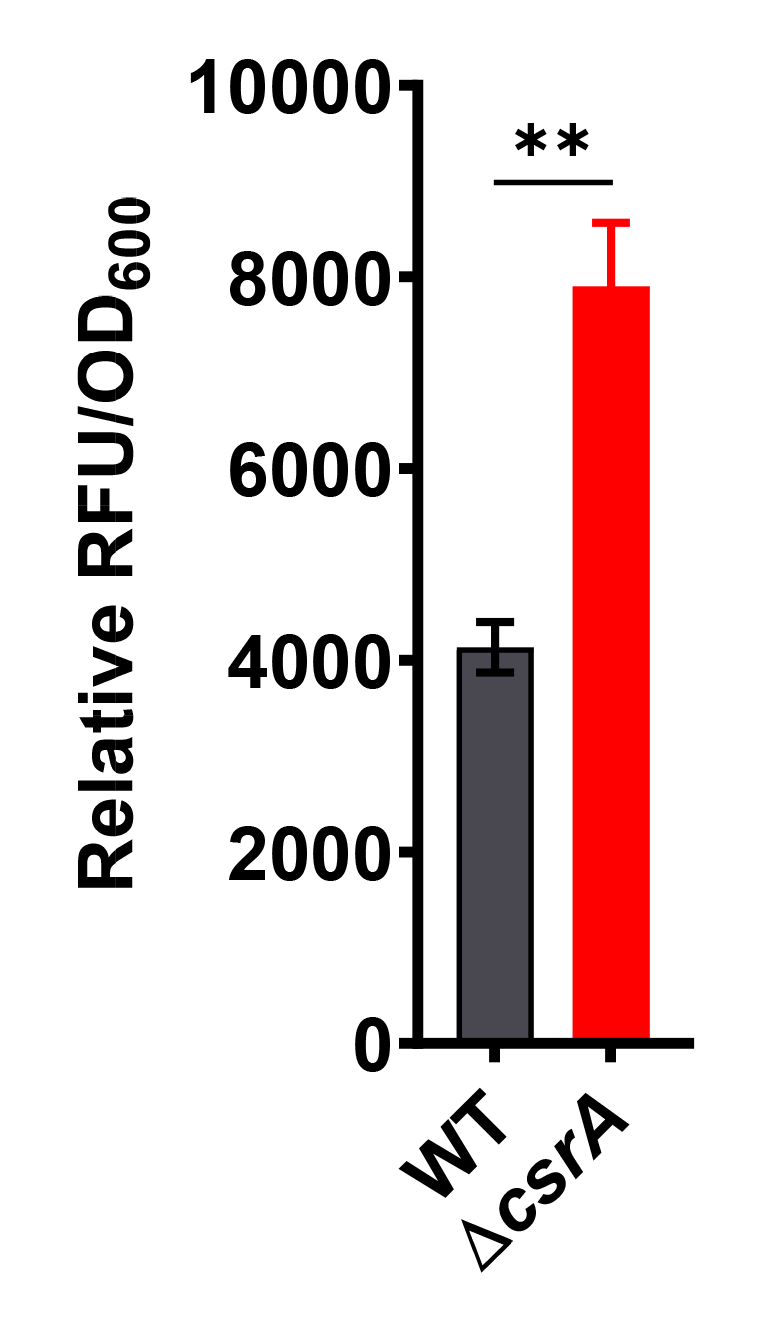

Supplement: FIG S2 [file mbio.01358-21-sf002.tif]

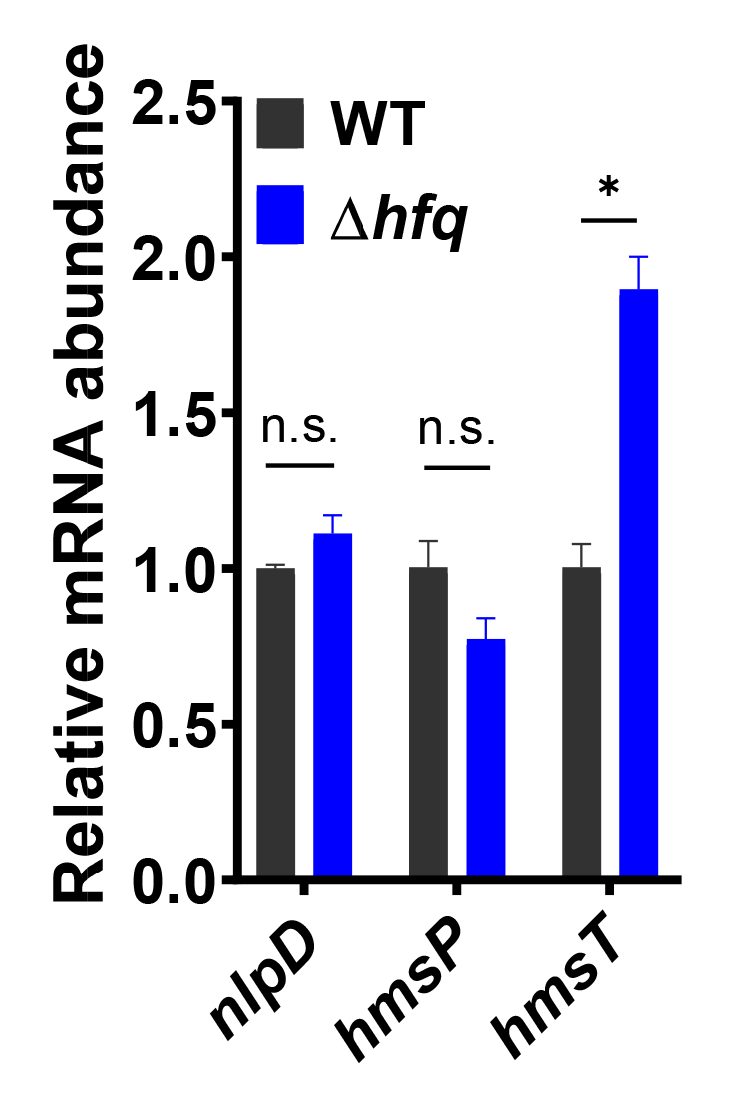

Supplement: FIG S3 [file mbio.01358-21-sf003.tif]

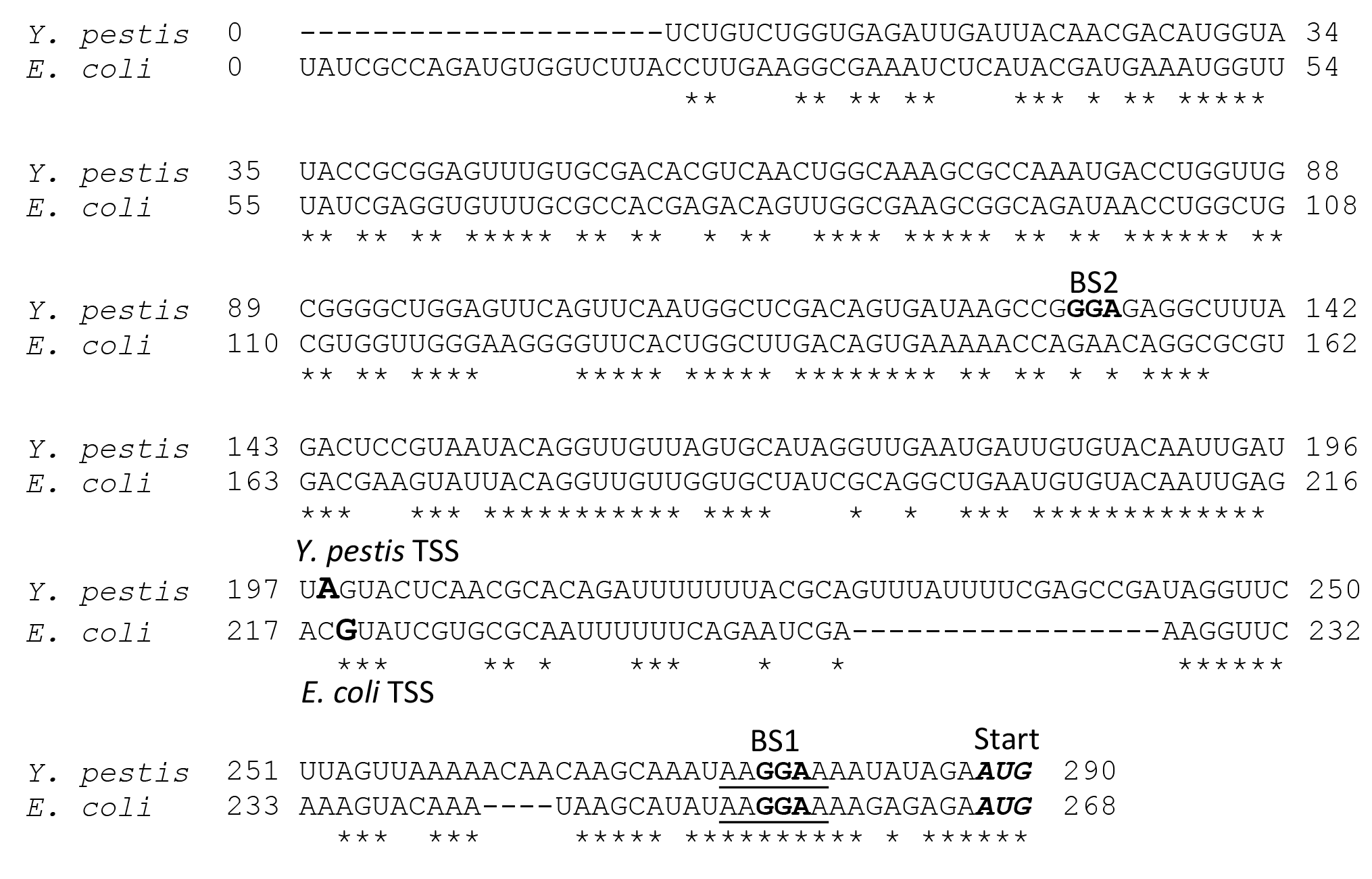

Supplement: FIG S4 [file mbio.01358-21-sf004.tif]
