# Supplementary material for: CsrA Enhances Cyclic-di-GMP Biosynthesis and Yersinia pestis Biofilm Blockage of the Flea Foregut by Alleviating Hfq-Dependent Repression of the hmsT mRNA
Source: mBio. 2021 Aug 3;12(4):e01358-21. doi: 10.1128/mBio.01358-21 (PMC8406273; doi:10.1128/mBio.01358-21)
Supplement: TABLE S1 [file mbio.01358-21-st001.docx]

**Table S1: Bacterial strains and plasmids used in this study**

| **Strain** | **Description** | **Reference** |
| --- | --- | --- |
| ***Y. pestis* KIM6+ strains** |  |  |
| WT | Pgm+ pCD1- pMT1+ pPCP1+, parental strain | [1] |
| Δ*csrA* pKD46 | ∆*csrA* 4:12 pKD46 | [1] |
| Δ*csrA* | ∆*csrA* 4:12 | This study |
| ∆*csrA*::*csrA* | ∆*csrA* 4:12::*att*Tn7::*csrA* | This study |
| ∆*hmsR* | Δ*hmsR::kan*^R^ | Bob Perry |
| ∆*hmsT* | Δ*hmsT::kan*^R^ | [2] |
| WT *gyrB-gfp* | WT pMWO78::*gyrB-gfpmut3.1* | This study |
| Δ*csrAgyrB-gfp* | Δ*csrA* pMWO78::*gyrB-gfpmut3.1* | This study |
| WT *flhdc-gfp* | WT pMWO78::*flhdc-gfpmut3.1* | This study |
| Δ*csrAflhdc-gfp* | Δ*csrA* pMWO78::*flhdc-gfpmut3.1* | This study |
| WT *hmsT-gfp* | WT pMWO78::*hmsT-gfpmut3.1* | This study |
| Δ*csrAhmsT-gfp* | Δ*csrA* pMWO78::*hmsT-gfpmut3.1* | This study |
| WT *hmsP-gfp* | WT pMWO78::*hmsP-gfpmut3.1* | This study |
| Δ*csrAhmsP-gfp* | Δ*csrA* pMWO78::*hmsP-gfpmut3.1* | This study |
| WT *hfq-gfp* | WT pMWO78::*hfq-gfpmut3.1* | This study |
| ∆*csrA hfq-gfp* | ∆*csrA* pMWO78::*hfq-gfpmut3.1* | This study |
| WT *hfq-gfp-short* | WT pMWO78::*hfq-gfpmut3.1 short* | This study |
| ∆*csrA hfq-gfp-short* | ∆*csrA* pMWO78::*hfq-gfpmut3.1 short* | This study |
| Δ*hfq* | Δ*hfq::kan*^R^ | [3] |
| ∆*csrA∆hfq* | deletion of *hfq* in ∆*csrA* 4:12 | This study |
| ∆*csrA∆hfq* pLG*hfq* | ∆*csrA∆hfq* pLG::*hfq* | This study |
|  |  |  |
| ***E.* *coli* strains** |  |  |
| S17lambda pir | TpR SmR recA, thi, pro, hsdR-M+RP4: 2-Tc:Mu: Km Tn7 λpir | Lab stock |
| BL21λ(DE3) pLysS | F–, *ompT*, *hsdS*_B_ (r_B_–, m_B_–), *dcm*, *gal*, λ(DE3), pLysS, Cm^R^ | Novagen |
| BL21λ(DE3) pLysS pET28A::*csrA-his6* | F–, *ompT*, *hsdS*_B_ (r_B_–, m_B_–), *dcm*, *gal*, λ(DE3), pLysS, Cm^R^, *csrA-his6* | This study |
|  |  |  |
| **Plasmid** | **Description** | **Reference** |
| pKD46 | Cb^R^ | [1] |
| pUC18R6KT-mini-Tn7T-Km | Amp^R^, Kan^R^; Cloning vector for Tn7 insertion | [4] |
| pUC18R6KT-mini-Tn7T-Km-*csrA* | Amp^R^, Kan^R^; Cloning vector for Tn7 insertion of *csrA* (-300 bp, +134 bp of *csrA*) | This study |
| pTN7S2 | Amp^R^; Encodes the TnsABC+D specific transposition pathway | [4] |
| pFLP3 | Amp^R^, Tc^R^; Source of Flp recombinase | [4] |
| pFU34 | Amp^R^; Source of *gfpmut3.1* used for reporter fusions | [5] |
| pMWO78 | Spec^R^; p15A; Contains the *tet* operator/promoter system | [6] |
| pMWO78::*flhDC-gfpmut3.1* | Spec^R^; *flhDC* (-194 bp, +27 bp of *flhDC*) | This study |
| pMWO78::*gyrB-gfpmut3.1* | Spec^R^; *gyrB* (-35 bp, +30 bp of *gyrb*) | This study |
| pMWO78::*hmsT*-*gfpmut3.1* | Spec^R^; *hmsT* (-57 bp, +27 bp of *hmsT*) | This study |
| pMWO78::*hmsP-gfpmut3.1* | Spec^r^; *hmsP* (-220 bp, +171 bp of *hmsP*) | This study |
| pMWO78::*hfq-gfpmut3.1* | Spec^R^; *hfq* (-177 bp, +27 bp of *hfq*) | This study |
| pMWO78::*hfq-gfpmut3.1 short* | Spec^R^; *hfq* (-115 bp, +27 bp of *hfq*) | This study |
| pET28A | Kan^R^; Nterm *his6*, Cterm *his6*, Cloning vector for protein expression | Novagen |
| pET28A::*csrA-his6* | Kan^R^; (+1 bp, +183 bp of *csrA*) fused to Cterm *his6* | This study |
| pJet1.2 | Amp^R^ | Thermo Fisher |
| pJet1.2::*hfq* UTR BS1mut | Amp^R^; hfq (-177 bp, +74 bp) with BS1 mutated to CC from GG | This study |
| pJet1.2::*hfq* UTR with BS2 mutation | Amp^R^; hfq (-177 bp, +74 bp) with BS2 mutated to CCC from GGA | This study |
| pJet1.2::*hfq* UTR with BS1/BS2 mutation | Amp^R^; hfq (-177 bp, +74 bp) with BS1 mutated to CC from GG and BS2 mutated to CCC from GGA | This study |
|  |  |  |
| pLG338 | Kan^R^, Tet^R^ | [7] |
| pLG*hfq* | pLG338::*hfq,* Kan^R^, Tet^R^; (-300 bp, +606 bp of *hfq*) | This study |

**References**

1. **Willias SP, Chauhan S, Lo C-C, Chain PSG, Motin VL.** 2015. CRP-Mediated Carbon Catabolite Regulation of *Yersinia pestis* Biofilm Formation Is Enhanced by the Carbon Storage Regulator Protein, CsrA. PLOS ONE **10:**e0135481.
2. **Jones HA, Lillard JW, and Perry RD.** 1999. HmsT, a Protein Essential for Expression of the Haemin Storage (Hms+) Phenotype of *Yersinia Pestis*. Microbiology 145, no. 8: 2117–28.
3. **Rempe KA, Hinz AK, Vadyvaloo V.** 2012. Hfq regulates biofilm gut blockage that facilitates flea-borne transmission of *Yersinia pestis*. J Bacteriol **194:**2036-2040
4. **Choi KH, Gaynor JB, White KG, Lopez C, Bosio CM, Karkhoff-Schweizer RR, Schweizer HP.** 2005. A Tn7-based broad-range bacterial cloning and expression system. Nat Methods **2:**443-448.
5. **Uliczka F, Pisano F, Kochut A, Opitz W, Herbst K, Stolz T, Dersch P.** 2011. Monitoring of Gene Expression in Bacteria during Infections Using an Adaptable Set of Bioluminescent, Fluorescent and Colorigenic Fusion Vectors. Plos One **6**:e20425.
6. **Obrist MW, Miller VL.2012.** Low copy expression vectors for use in *Yersinia sp*. and related organisms. Plasmid **68:**33-42.
7. **Stoker NG, Fairweather NF, Spratt BG.** 1982. Versatile low-copy-number plasmid vectors for cloning in *Escherichia coli*. Gene **18:**335-341.
