# Supplementary material for: CsrA Enhances Cyclic-di-GMP Biosynthesis and Yersinia pestis Biofilm Blockage of the Flea Foregut by Alleviating Hfq-Dependent Repression of the hmsT mRNA
Source: mBio. 2021 Aug 3;12(4):e01358-21. doi: 10.1128/mBio.01358-21 (PMC8406273; doi:10.1128/mBio.01358-21)
Supplement: TABLE S2 [file mbio.01358-21-st002.docx]

**Table S2: Primers used in this study**

| **Description** | **Sequence** | **Reference** |
| --- | --- | --- |
|  |  |  |
| **Mutant and complementation strain construction** | | |
| *csrA* F | TTGCCAGCGGCATTAGCCAG | This study |
| *csrA* R | TCTGAGCGGTAGTGACTGGGCAGAC | This study |
| M13 F | GTAAAACGACGGCCAG | Thermo Fisher |
| M13 R | CAGGAAACAGCTATGAC | Thermo Fisher |
| P_Tn7L_ | ATTAGCTTACGACGCTACACCC | [1] |
| P_Tn7R_ | ACAGCATAACTGGACTGATTTC | [1] |
| P_PstSUp2_ | GCTATACGTGTTTGCTGATCAAGATGC | [1] |
| P*_glmS_*_2-DN_ | ACGCCACCGGAAGAACCGATACCT | [1] |
| *hfq* deletion F | TGGCAAAGCGCCAAATGACCTGG | This study |
| *hfq* deletion R | CAAACGGGTCGCGATATGACGC | This study |
| *hfq* complementation F | TTCGGGTGAAACCTTACCTTACCG | (2) |
| *hfq* complementation F | GCGCAGATCCTCTGTGTCTTTGTT | (2) |
|  |  |  |
| **qRT-PCR** | | |
| *gyrB* F | TCGCCGTGAAGGTAAAGTTC | Lab stock |
| *gyrB* R | TTTGCTCAGTTTCACCCACC | Lab stock |
| *hmsT* F | CAAAGAACGACACGGGCAGAAATAAC | [3] |
| *hmsT* R | TGCCCAAAGTAGACCGATGAGGATTG | [3] |
| *hmsP* F | ATGAGTGAAGCCCAGCACCAACAG | [3] |
| *hmsP* R | ACCTCTGGCTAACACGGCAAATTCAG | [3] |
| *hfq* F | TGCAGGGCCAAGTTGAGTCT | This study |
| *hfq* R | ATGCGAAACCGGACGAGAAG | This study |
| *nlpD* F | ATATCGCTTGGATAACAGGAAATGAC | Lab stock |
| *nlpD* R | TTGAATAGATTGCCCCACATTCAGG | Lab stock |
|  |  |  |
| **Translational fusion reporters** | | |
| 5' UTR *flhDC* | CACGACGAATTCGAGCGAATTTTGTAAAGTGGCTC | This study |
| 3' *flhDC* | GTTCTTCTCCTTTACGCATATGTTTGAGTAATTCAGACGTACTCATC | This study |
| 5' UTR *gyrB* | CACATCGAATTCTCAACCACAGGATTAAAAATGAGCG | This study |
| 3' *gyrB* | GTGAAAAGTTCTTCTCCTTTACGCATACTTGAGGAGTCATAAGTATTCGACAT | This study |
| 5' UTR *hmsT* | GAGCCAGAATTCGTCGGAATAAATACCTCACGAGG | This study |
| 3' *hmsT* | AAAAGTTCTTCTCCTTTACGCATGCTATTCATATTCAATTTACTCTGCATAATATCG | This study |
| 5' UTR *hmsP* | AGCTGTGAATTCGGTCAGGGGTGGTTACAGC | This study |
| 3' *hmsP* | AGTGAAAAGTTCTTCTCCTTTACGCATAATCGTTAATGAGCGCCTTACCCGCA | This study |
| 5' UTR *hfq* | GATCGGGAATTCGTTGAATGATTGTGTACAATTGATTAG | This study |
| 5' UTR *hfq short* | GATCGGGAATTCGGGCTCGACAGTGATAAGCC | This study |
| 3' *hfq* | AAAAGTTCTTCTCCTTTACGCATATAAATAGAAACCGGAACCCGT | This study |
| 5' *gfp* for *flhDC* | TACGTCTGAATTACTCAAACATATGCGTAAAGGAGAAGAACTTTTCAC | This study |
| 5' *gfp* for *gyrB* | ATGTCGAATACTTATGACTCCTCAAGTATGCGTAAAGGAGAAGAACTTTTCAC | This study |
| 5' *gfp* for *hmsT* | GCAGAGTAAATTGAATATGAATAGCATGCGTAAAGGAGAAGAACTTTTCAC | This study |
| 5' *gfp* for *hmsP* | TGCGGGTAAGGCGCTCATTAACGATTATGCGTAAAGGAGAAGAACTTTTCACT | This study |
| 5' *gfp* for *hfq* | GGCTAAGGGGCAATCTTTGCAAGATATGCGTAAAGGAGAAGAACTTTTCAC | This study |
| 3' *gfp* | TTATTTGTATAGTTCATCCATGCCATGTGTAATCC | This study |
| MWO-F | CCCATTATTATCATGACATTAAC | This study |
| MWO-075-R | CATCTTCCAGGAAATCTCCG | This study |
|  |  |  |
| **CsrA-his tag construct** | | |
| 5' *csrA* for pET28A | AGCGCCCCATGGCAATGCTTATTCTGACTCGT | This study |
| 3' *csrA* for pET28A | CGCCGCCTCGAGGTAAGTCGTCGGTTGAGAC | This study |
|  |  |  |
| **EMSA probes** | | |
| T7 5' *flhDC* | GTAATACGACTCACTATAGGAGCGAATTTTGTAAAGTGGC | [4] |
| 3' *flhD*C | CCGTTGAGCTAAAAGTAAATATG | [4] |
| T7 5' *hns* | GTAATACGACTCACTATAGGCACTCTATTATTATCCAGAC | [4] |
| 3' *hns* | GCAGTTCATTTGGATCAATAC | [4] |
| T7 5' *hmsT* | GTAATACGACTCACTATAGGGTGAACGACATCATGATGACAGG | This study |
| 3' *hmsT* | GCAGTAAAGAGAGTCGATGC | This study |
| T7 5' *hmsP* | GTAATACGACTCACTATAGGGTCAGGGGTGGTTACAG | This study |
| 3' *hmsP* | TAATCGTTAATGAGCGCCTTACC | This study |
| T7 5' *hfq* | GTAATACGACTCACTATAGGGCTCGACAGTGATAAGCCG | This study |
| T7 5' *hfq short* | GTAATACGACTCACTATAGGGTTGAATGATTGTGTACAATTGATTAG | This study |
| T7 5' *hfq* BS2 mut | GTAATACGACTCACTATAGGGCTCGACAGTGATAAGCCGCCCGAGGCTTT | This study |
| 3' *hfq* | TAAATAGAAACCGGAACCCGTTC | This study |
| *hfq* 5' BS2 mut | CCCGAGGCTTTAGACTCCGTAATACAGG | This study |
| *hfq* 3' BS2 mut | CGGCTTATCACTGTCGAGCCCTATA | This study |
| *hfq* 5' BS1 | AAAATATAGAATGGCTAAGGGGC | This study |
| *hfq* 5' BS1 mut | CCTTATTTGCTTGTTGTTTTTAACTAAGAAC | This study |

Grey and underlined font indicates substitution mutations.

**References**

1. **Choi KH, Gaynor JB, White KG, Lopez C, Bosio CM, Karkhoff-Schweizer RR, Schweizer HP.** 2005. A Tn7-based broad-range bacterial cloning and expression system. Nat Methods **2:**443-448.
2. **Rempe KA, Hinz AK, Vadyvaloo V.** 2012. Hfq regulates biofilm gut blockage that facilitates flea-borne transmission of *Yersinia pestis*. J Bacteriol **194:**2036-2040
3. **Bellows LE, Koestler BJ, Karaba SM, Waters CM, Lathem WW.** 2012. Hfq-dependent, coordinate control of cyclic diguanylate synthesis and catabolism in the plague pathogen *Yersinia pestis*. Mol Microbiol **86:**661-674.

4. **Heroven AK, Bohme K, Rohde M, Dersch P.** 2008. A Csr-type regulatory system, including small non-coding RNAs, regulates the global virulence regulator RovA of *Yersinia pseudotuberculosis* through RovM. Molecular Microbiology **68:**1179-1195.
